# Thylakoid localized bestrophin-like proteins are essential for the CO_2_ concentrating mechanism of *Chlamydomonas reinhardtii*

**DOI:** 10.1073/pnas.1909706116

**Published:** 2019-08-07

**Authors:** Ananya Mukherjee, Chun Sing Lau, Charlotte E. Walker, Ashwani K. Rai, Camille I. Prejean, Gary Yates, Thomas Emrich-Mills, Spencer G. Lemoine, David J. Vinyard, Luke C. M. Mackinder, James V. Moroney

**Affiliations:** ^a^Department of Biological Sciences, Louisiana State University, Baton Rouge, LA 70803;; ^b^Department of Biology, University of York, Heslington, York YO10 5DD, United Kingdom

**Keywords:** *Chlamydomonas*, CO_2_ concentrating mechanism, bicarbonate transport, photosynthesis, chloroplast thylakoid

## Abstract

The green alga *Chlamydomonas reinhardtii* possesses a CO_2_ concentrating mechanism (CCM) that helps in successful acclimation to low CO_2_ conditions. Current models of the CCM postulate that a series of ion transporters bring HCO_3_^−^ from outside the cell to the thylakoid lumen, where the carbonic anhydrase 3 (CAH3) dehydrates accumulated HCO_3_^−^ to CO_2_, raising the CO_2_ concentration for Ribulose bisphosphate carboxylase/oxygenase (Rubisco). Previously, HCO_3_^−^ transporters have been identified at both the plasma membrane and the chloroplast envelope, but the transporter thought to be on the thylakoid membrane has not been identified. Three paralogous genes (*BST1*, *BST2*, and *BST3*) belonging to the bestrophin family have been found to be up-regulated in low CO_2_ conditions, and their expression is controlled by CIA5, a transcription factor that controls many CCM genes. YFP fusions demonstrate that all 3 proteins are located on the thylakoid membrane, and interactome studies indicate that they might associate with chloroplast CCM components. A single mutant defective in *BST3* has near-normal growth on low CO_2_, indicating that the 3 bestrophin-like proteins may have redundant functions. Therefore, an RNA interference (RNAi) approach was adopted to reduce the expression of all 3 genes at once. RNAi mutants with reduced expression of *BST1*–*3* were unable to grow at low CO_2_ concentrations, exhibited a reduced affinity to inorganic carbon (C_i_) compared with the wild-type cells, and showed reduced C_i_ uptake. We propose that these bestrophin-like proteins are essential components of the CCM that deliver HCO_3_^−^ accumulated in the chloroplast stroma to CAH3 inside the thylakoid lumen.

## Significance

Models of the CO_2_ concentrating mechanism (CCM) of green algae and diatoms postulate that chloroplast CO_2_ is generated from HCO_3_^−^ brought into the acidic thylakoid lumen and converted to CO_2_ by specific thylakoid carbonic anhydrases. However, the identity of the transporter required for thylakoid HCO_3_^−^ uptake has remained elusive. In this work, 3 bestrophin-like proteins, BST1–3, located on the thylakoid membrane have been found to be essential to the CCM of *Chlamydomonas*. Reduction in expression of BST1–3 markedly reduced the inorganic carbon affinity of the alga. These proteins are prime candidates to be thylakoid HCO_3_^−^ transporters, a critical currently missing step of the CCM required for future engineering efforts of the *Chlamydomonas* CCM into plants to improve photosynthesis.

Aquatic photosynthetic organisms, which account for close to 50% of the world’s carbon fixation (1), face several challenges in carrying out efficient photosynthesis. Limitations include the slow diffusive rate of gases in water, fluctuations in pH, and the slow interconversion of inorganic carbon (C_i_) forms. Thus, most aquatic autotrophs have developed an adaptation called the CO_2_ concentrating mechanism (CCM) that increases the concentration of CO_2_ around Ribulose bisphosphate carboxylase/oxygenase (Rubisco) to increase its carboxylase activity. Aside from Rubisco’s slow rate of catalysis, O_2_ can compete with CO_2_ for the active site of the enzyme, resulting in the wasteful process of photorespiration (2). Since CO_2_ and O_2_ are competitive substrates, the CCM reduces photorespiration and increases photosynthetic efficiency.

The CCM of the unicellular green alga *Chlamydomonas reinhardtii* (hereafter referred to as *Chlamydomonas*) has a number of bicarbonate (HCO_3_^−^) transporters that help increase the HCO_3_^−^ concentration in the chloroplast stroma relative to the external HCO_3_^−^ concentration. These transporters are located on the plasma membrane (LCI1 and HLA3) as well as the chloroplast envelope (NAR1.2/LCIA). Loss of any one of these transporters reduces the ability of the cell to accumulate HCO_3_^−^ at high external pH (3, 4). In addition, Rubisco is tightly packaged in a microcompartment of the chloroplast called the pyrenoid (5–7). Finally, carbonic anhydrase 3 (CAH3), located in the lumen of pyrenoid-traversing thylakoids, converts the accumulated HCO_3_^−^ to CO_2_ near the site of Rubisco (8, 9), increasing photosynthetic and growth rates at otherwise growth-limiting CO_2_ levels.

Carbonic anhydrases play an essential role in the *Chlamydomonas* CCM (10). The loss of CAH3 results in cells that cannot grow on air levels of CO_2_, even though these mutants tend to overaccumulate HCO_3_^−^ (11). *Chlamydomonas* CCM models propose that mutants missing CAH3 accumulate the HCO_3_^−^ brought into the chloroplast by the transport proteins but cannot convert that HCO_3_^−^ to CO_2_, the actual substrate of Rubisco (12, 13). These CCM models postulate that the pH gradient across the thylakoid membrane in the light helps drive the conversion of HCO_3_^−^ to CO_2_. The apparent acid dissociation constant (pK_a_) of the interconversion of HCO_3_^−^ and CO_2_ is about 6.4, with the chloroplast stoma having a pH close to 8 in the light and the thylakoid lumen having a pH close to 5.7 under low CO_2_ concentrations (14). Therefore, as HCO_3_^−^ is brought from the stroma to the thylakoid lumen, it goes from an environment favoring HCO_3_^−^ to one favoring CO_2_. Therefore, the acidification of the thylakoid lumen is important to the functioning of the CCM.

The CCM models also proposed the presence of a thylakoid HCO_3_^−^ transporter that brings in HCO_3_^−^ from the stroma to the lumen for dehydration by CAH3 (12, 13). In a recent interactome study, the CCM complex LCIB/LCIC is shown to interact with the bestrophin-like proteins encoded by Cre16.g662600 and Cre16.g663450 (15). These proteins were also shown to interact with each other and another bestrophin-like protein encoded by Cre16.g663400 (15). All 3 genes were found to be up-regulated in low CO_2_ conditions in a transcriptomic study showing they belonged to a cluster of genes that had increased expression in low CO_2_ and were controlled by CIA5 (16). Bestrophins are typically chloride channels, including the *Arabidopsis* bestrophin-like protein AtVCCN1 (17). However, they have also been shown to transport a range of anions, with some showing high HCO_3_^−^ permeability (18). The interactome study also putatively localizes these bestrophin-like proteins to the thylakoid membrane, which makes them promising candidates to be the thylakoid HCO_3_^−^ transporter in the CCM of *Chlamydomonas*.

In the present study, we investigate the role of these 3 proteins using an RNA interference (RNAi) approach to knock down the expression of all 3 genes. This approach was feasible as the 3 genes are extremely similar at the DNA sequence level. Knockdown mutants with low expression of all 3 genes grow poorly in limiting CO_2_ conditions, exhibit a poor affinity for external C_i_, and have a severely reduced ability to accumulate HCO_3_^−^. This study sheds light on the intracellular location and function of these bestrophin-like proteins in the CCM of *Chlamydomonas*.

## Results

### *Chlamydomonas* Has 3 Very Similar Bestrophin-Like Proteins on the Thylakoid Membrane.

**Fig. 1.**
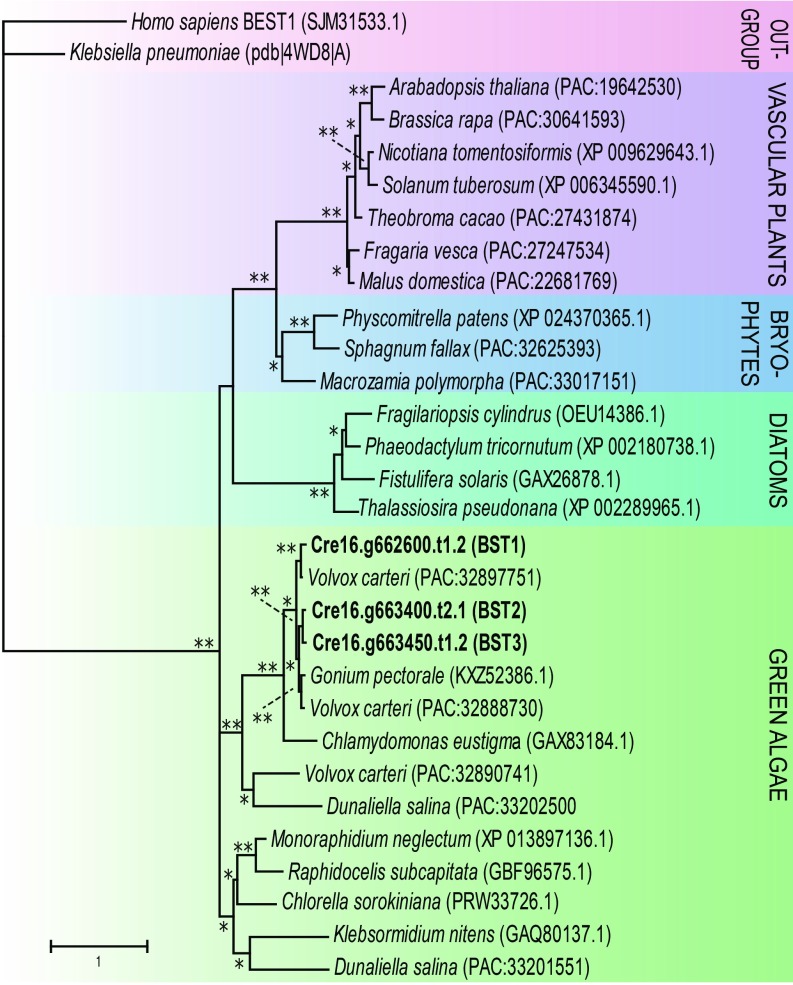
Phylogenetic analysis of *Chlamydomonas* bestrophin-like proteins BST1–3. The evolutionary history of *Chlamydomonas* bestrophin-like proteins BST1–3 was inferred by using the maximum likelihood method based on the Le and Gascuel (37) model with discrete Gamma distribution (5 categories) and 500 bootstrap replicates. The tree is drawn to scale, with branch lengths measured in the number of substitutions per site. *Bootstrap value ≥ 50, **Bootstrap value ≥ 90.

*BST1* (Cre16.g662600), *BST2* (Cre16.g663400), and *BST3* (Cre16.g663450) (collectively *BST1*–*3*) are paralogous bestrophin-like genes located within a 130-kilobase pair (kbp) region on the 16th chromosome of *Chlamydomonas*. Phylogenetic analyses revealed that bestrophin-like proteins are found in a diverse variety of photosynthetic organisms (Fig. 1), including vascular plants, nonvascular plants, and diatoms, with the homologs with the highest sequence identity to BST1–3 found in algae. The amino acid sequences encoded by these genes were analyzed in TMHMM, which predicted that BST1–3 are membrane proteins having 4 predicted transmembrane domains each. Further analysis using PredAlgo predicted that each BST protein had a chloroplast transit peptide and was likely to be a chloroplast membrane protein. BST1 was annotated as a bestrophin-like protein in Phytozome (version 12.1), and *BST2* and *BST3* were previously reported as *LCI11* by Fang et al. (16). An alignment between the 3 *Chlamydomonas* bestrophin-like proteins showed that the proteins are >80% identical to one another (*SI Appendix*, Fig. S1). There are 7 more genes annotated as encoding bestrophin-like proteins in the *Chlamydomonas* genome, but they share less than 50% identity to BST1–3. Sequence alignment of BST1–3 with human Bestrophin 1 (BEST1) showed low sequence identity between BEST1 and BST1–3 (21 to 23%; *SI Appendix*, Fig. S1). The most similar protein in terrestrial plants, the thylakoid localized AtVCCN1 protein of *Arabidopsis* (17), has approximately a 30% sequence identity with BST1–3. To further explore the potential structure and function of BST1–3, we did homology modeling using SWISS-MODEL (19). Structural studies show that human and *Klebsiella pneumoniae* bestrophins are pentameric, and modeling of BST1 in a pentameric assembly is of high confidence (*SI Appendix*, Fig. S2*A*). The highest ranking template identified by SWISS-MODEL for BST1–3 was *K. pneumoniae* bestrophin. BST1–3 contain nonpolar residues along their selective pore that are conserved in proteins of the bestrophin family and are involved in anion transport (20) (*SI Appendix*, Fig. S2*B*). The entry pocket of BST1 has a predominantly neutral/negative electrostatic potential, and the selective pore is positively charged, supporting the hypothesis that BST1–3 transport negatively charged ions (19, 21) (*SI Appendix*, Fig. S2 *C* and *D*), as does AtVCCN1 in *Arabidopsis* (17).

### BST1–3 Are Up-Regulated under Low CO_2_ Growth Conditions and Localized to the Thylakoid.

**Fig. 2.**
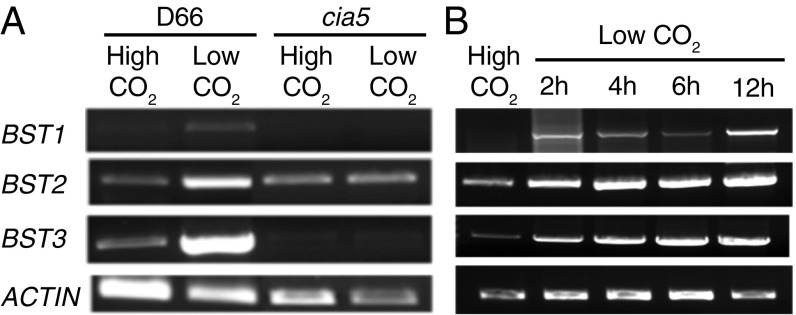
Transcript analysis of *BST1*–*3*. (*A*) Semiquantitative RT-PCR showing *BST1*–*3* accumulation in ambient CO_2_ (0.04% CO_2_) vs. high CO_2_ (5% [vol/vol] CO_2_ in air) in D66 and *cia5* cells. (*B*) Semiquantitative RT-PCR time course showing the expression of *BST1*–*3* in complementary DNA obtained from high CO_2_ (5% CO_2_ [vol/vol] in air) and in cells switched to ambient CO_2_ (0.04% CO_2_) for the indicated times. Actin has been used as a loading control.

Semiquantitative RT-PCR (Fig. 2*A*) was performed using complementary DNA isolated from strains D66 and *cia5* grown under high CO_2_ or ambient CO_2_ conditions. For this work, we have used 5% CO_2_ (vol/vol) in air as high CO_2_, 0.04% as ambient CO_2_, and <0.02% as low CO_2._ D66 is the wild-type strain for these studies, and *cia5* is missing the CCM1 protein, which is required for the induction of the CCM in *Chlamydomonas* (22). This work demonstrated that all 3 *BST* genes were up-regulated under ambient CO_2_ growth conditions in D66 and that this up-regulation was not observed in *cia5* (*SI Appendix*, Fig. S3*A*). In addition, the *cia5* mutant exhibited severely reduced expression of *BST1* and *BST3* under both CO_2_ conditions, a transcriptional pattern observed with other CCM genes. *BST2* transcript levels in *cia5* cells showed reduced induction in ambient CO_2_ when compared with D66 cells, where *BST2* transcript levels increase in ambient CO_2_ conditions. A time course study of the expression of these 3 genes during induction of the CCM was done by transferring high CO_2_-grown cells to ambient CO_2_ levels for 2 to 12 h (Fig. 2*B* and *SI Appendix*, Fig. S3*B*)_._ All 3 genes had increased transcript levels within 2 h after the switch to low CO_2_, and these elevated levels of expression continued until at least 12 h after induction. *BST1* had a lower level of expression than *BST2* or *BST3* (Fig. 2).

**Fig. 3.**
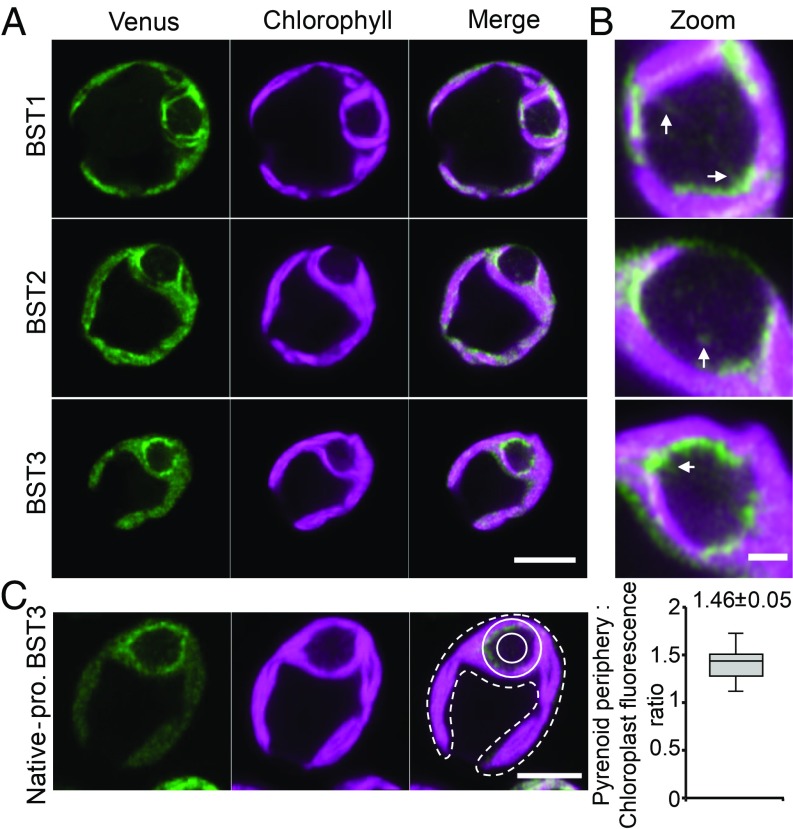
Localization of BST1–3. (*A*) Confocal microscopy of BST1–3 proteins fused with Venus (green) and driven by the constitutive *PSAD* promoter. Chlorophyll autofluorescence is shown in magenta. (Scale bar, 5 μm.) (*B*) Zoomed-in images of BST1–3 pyrenoids shown in *A*. Arrows highlight where Venus fluorescence is seen overlapping with chlorophyll fluorescence in the pyrenoid matrix. (Scale bar, 1 μm.) (*C*) Localization and quantification of BST3 distribution under its native promoter. The ratio of fluorescence intensity at the pyrenoid periphery (solid line region) and chloroplast (dotted line region) was quantified. The value above the plot denotes the mean ± SE (*n* = 23). (Scale bar, 4 μm.)

To determine the localization of these 3 BST-like proteins in *Chlamydomonas*, fluorescent protein fusions were constructed linking Venus to the C terminus of each BST protein. All 3 BST-like proteins localized to the thylakoid membranes of the chloroplast (Fig. 3*A*), and this localization visibly extended into the thylakoid tubules of the pyrenoid (Fig. 3*B*). The localization studies visually showed that BST1, BST2, and BST3 were preferentially concentrated near the pyrenoid (Fig. 3*B*). To confirm that expression using the constitutive *PSAD* promoter was not affecting localization or pyrenoid periphery enrichment, we constructed a BST3-Venus line with the BST3 gene under its native promoter. This line showed the same localization pattern as BST3 under the constitutive *PSAD* promoter (Fig. 3*C*), and quantification of enrichment showed a 1.46-fold enrichment (*P <* 0.01, Student’s paired *t* test) around the pyrenoid relative to the rest of the chloroplast. Thus, BST1, BST2, and BST3 are thylakoid localized anion transporters enriched at the pyrenoid periphery that are expressed coordinately with the expression of other *Chlamydomonas* CCM proteins.

### Reduction of BST1–3 Expression Results in Cells that Grow Slowly under Low CO_2_ Conditions.

A BST3 knockout (*bst3*) was obtained from the *Chlamydomonas* Library Project (CLiP) mutant collection (23) with a paromomycin insert in the last exon of the *bst3* gene (*SI Appendix*, Fig. S4*A*). The BST3 transcript was not detected in *bst3* (*SI Appendix*, Fig. S4*B*), and the BST3 protein was absent (*SI Appendix*, Fig. S4*C*). We observed a weak growth difference for this strain as compared with wild-type cells under ambient CO_2_ (*SI Appendix*, Fig. S5 *A* and *B*), but no clear phenotype on plates at pH 7 or pH 8.4 at 100 μmol of photons per m^−2^⋅s^−1^ at low CO_2_ (*SI Appendix*, Fig. S5*C*). However, there was no significant difference in C_i_ affinity between wild type and *bst3* grown at ambient CO_2_ (*SI Appendix*, Fig. S5*D*), and C_i_ uptake by *bst3* was only slightly lower than wild type (*SI Appendix*, Fig. S5 *E* and *F*). This led us to think that BST1 or BST2 function might be redundant with BST3 and that the expression of all 3 genes must be reduced to determine their physiological role(s). Therefore, to elucidate the function of *BST1*–*3*, RNAi constructs complementary to regions of identity among *BST1*–*3* were designed (*SI Appendix*, Table S1). The D66 strain was transformed with these constructs, and colonies were kept at high CO_2_. Colonies were then screened for growth on high CO_2_ versus low CO_2_, and *BST1*–*3* expression was quantified using RT-qPCR. Three independent colonies from 2 different transformations were chosen for further study and designated as *bsti-1*, *bsti-2*, and *bsti-3* (BST RNAi triple-knockdown lines 1, 2, and 3).

**Fig. 4.**
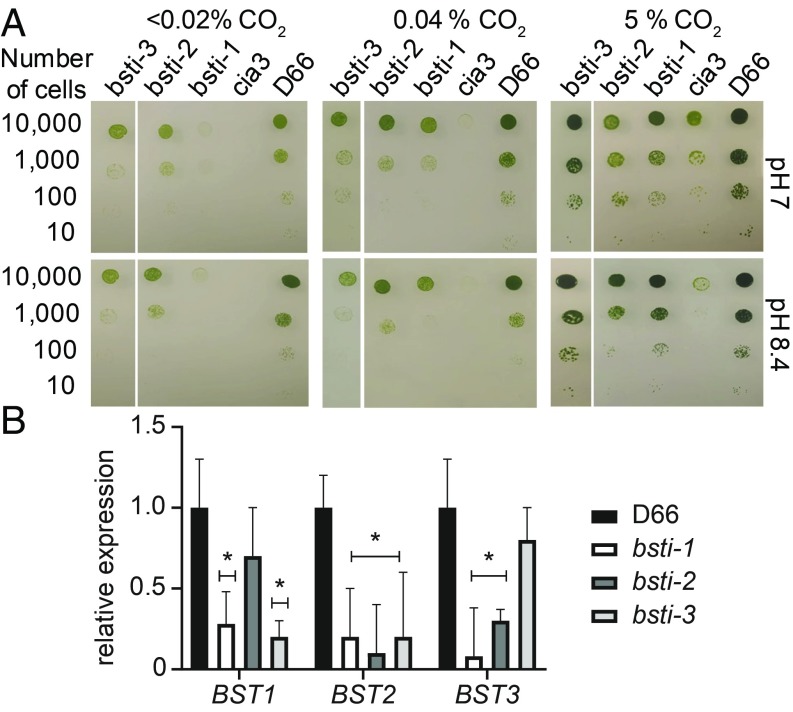
Growth of *bsti1*–*3* triple-knockdown RNAi lines and relative expression of *BST1*–*3* in the triple-knockdown lines. (*A*) Spot tests showing growth of D66, *cia3*, and *bsti1*–*3*. Cells were diluted to 6.6 × 10^5^ cells per milliliter, followed by 1:10 serial dilution 3 times to compare growth in low CO_2_ (<0.02% CO_2_), ambient CO_2_ (0.04% CO_2_), and high CO_2_ (5% CO_2_ [vol/vol] in air) at pH 7 and pH 8.4. Cells were grown for 6 d. The *CAH3* mutant, *cia3*, was included as a CCM-deficient control. (*B*) RT-qPCR shows that the expression of all 3 *BST* genes in the triple-knockdown lines is reduced when compared with their expression levels seen in D66. D66 and *bsti-1*, *bsti-2*, and *bsti-3* were acclimated to air levels of CO_2_ for 12 h before harvesting the RNA. *P < 0.05 by Student *t* test.

The growth of *bsti-1*, *bsti-2*, and *bsti-3* on high and low CO_2_ was compared with D66 and the *CAH3* knockout mutant, *cia3* (Fig. 4*A*). In low CO_2_, *bsti-1* showed severely reduced growth that was further exacerbated at high pH, resembling the growth of *cia3* (Fig. 4*A*). The *bsti-2* and *bsti-3* also grew more slowly than wild-type cells, but better than *bsti-1*. However, at high CO_2_, the growth of all 3 strains was comparable to wild type. RT-qPCR showed that *bsti-1* had significantly reduced expression of *BST1*, *BST2*, and *BST3* compared with D66 (Fig. 4*B*), and *bsti-2* and *bsti-3* had a more moderate knockdown of expression of the 3 genes. To see if reduced transcript levels resulted in decreased protein abundance, we checked BST3 protein levels in the knockdown lines. All showed reduced levels relative to D66, although this was only significant for *bsti-2* and *bsti-3* (*P <* 0.05, Student’s *t* test; *SI Appendix*, Fig. S6*A*). Thus, the BSTs are required for wild-type–like growth of *Chlamydomonas* under low CO_2_ conditions.

### Reduction of BST1–3 Expression Also Results in Cells that Have a Reduced Capacity to Accumulate Inorganic Carbon.

**Fig. 5.**
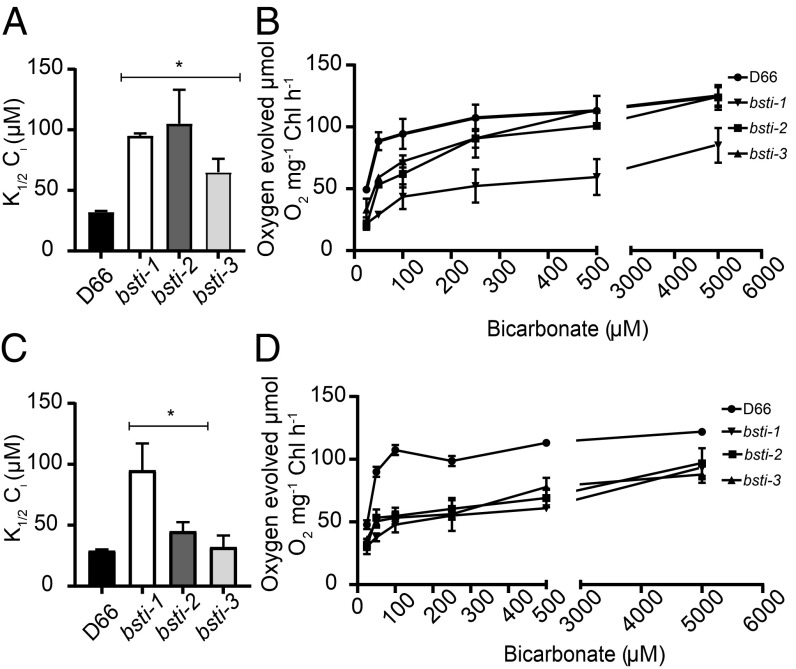
Photosynthetic oxygen evolution activity of *bst1*–*3* RNAi lines and D66. C_i_ affinity was estimated for *bsti1*–*3* and D66 acclimated to ambient CO_2_ for 12 h at pH 8.4 (*A* and *B*) and for *bsti1*–*3* and D66 at pH 7.8 (*C* and *D*). Oxygen evolving activity was measured at the indicated pH, and the K_1/2_(C_i_) values were calculated from the O_2_ evolution versus C_i_ curves. Triplicate runs were made at each C_i_ concentration. The differences in K_1/2_(C_i_) were significant (*P < 0.05 by Student’s *t*-test). At pH 7.8, the maximum velocity (V_max_) of D66 is 121 μmol of O_2_ per milligram of chlorophyll (Chl) per hour (O_2_ mg^−1^ Chl h^−1^), the V_max_ of *bsti-1* is 105 μmol of O_2_ mg^−1^ Chl h^−1^, the V_max_ of *bsti-2* is 87 μmol of O_2_ mg^−1^ Chl h^−1^, and the V_max_ of *bsti-3* is 90 μmol of O_2_ mg^−1^ Chl h^−1^. At pH 8.4, the V_max_ of D66 is 124 μmol of O_2_ mg^−1^ Chl h^−1^, the V_max_ of *bsti-1* is 85.5 μmol of O_2_ mg^−1^ Chl h^−1^, the V_max_ of *bsti-2* is 124 μmol of O_2_ mg^−1^ Chl h^−1^, and the V_max_ of *bsti-3* is 123 μmol of O_2_ mg^−1^ Chl h^−1^. Error bars indicate SD.

Two characteristics of algal cells with a CCM are a very high affinity for C_i_ and the ability to accumulate C_i_ to levels higher than can be obtained by diffusion. The *bsti-1*, *bsti-2*, and *bsti-3* acclimated to ambient CO_2_ exhibited a lower affinity for C_i_ as judged by their measured C_i_ concentration needed for half-maximum oxygen evolution [K_1/2_(C_i_)] (Fig. 5). When grown at high CO_2_, *bsti1*–*3* and D66 exhibited similar C_i_ affinities (*SI Appendix*, Fig. S6*B*). These results indicate that the expression of *BST1*–*3* is required for optimal C_i_ affinity when cells are grown on ambient levels of CO_2_. At pH 8.4, the K_1/2_(C_i_) values for *bsti1*–*3* are elevated in sharp contrast to a low K_1/2_(C_i_) for D66 (Fig. 5 *A* and *B*). At the higher pH of 8.4, the predominant C_i_ species in the medium would be HCO_3_^−^. Thus, the increased affinity of the cells for C_i_ reflects their ability to actively take up and utilize HCO_3_^−^. For *bsti-1*, where the expression of all 3 BST genes is between 60 and 90% reduced, there is a reduced C_i_ affinity at both pH 8.4 (Fig. 5 *A* and *B*) and pH 7.8 (Fig. 5 *C* and *D*). In contrast, *bst3*, the mutant missing only BST3, the difference in C_i_ affinity with wild type (*SI Appendix*, Fig. S2*B*) is much smaller. Thus, we can conclude that BST1–3 are necessary components of the CCM of *Chlamydomonas*.

**Fig. 6.**
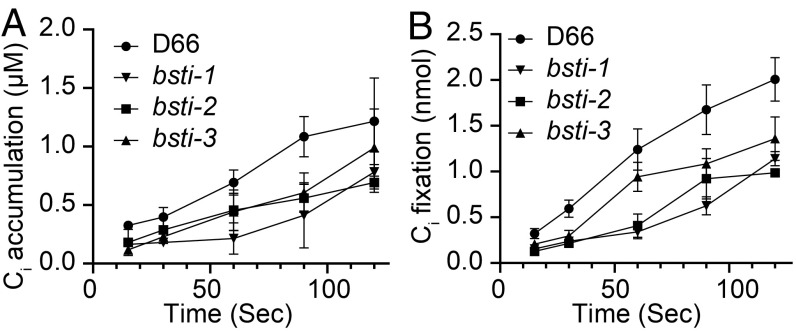
C_i_ uptake of D66 and *bsti1-3*. C_i_ uptake and C_i_ accumulation were measured in D66 and *bsti1-3* using the silicone oil uptake method (*Materials and Methods*). Cells were grown in high CO_2_ and then acclimated to ambient CO_2_ for 12 h prior to the assays. Cells were harvested and depleted of endogenous C_i_ before running the assays. A time course of intracellular C_i_ accumulation (*A*) and CO_2_ fixation (*B*) is shown for pH 8.4. Triplicate samples were run for each time point. The added H^14^CO_3_^−^ concentration was 50 μM.

C_i_ uptake activity was measured in D66, *bsti-1*, *bsti-2*, and *bsti-3* to evaluate the importance of BST1–3 in accumulation and fixation of C_i_. Ambient CO_2_-acclimated *bsti-1* had a notably lower accumulation and fixation of ^14^C_i_ compared with D66 at pH 8.4 (Fig. 6), and *bsti-2* and *bsti-3* also had inhibited ^14^C uptake and fixation, although not as reduced as *bsti-1* (Fig. 6). The most severely affected mutant, *bsti-1,* accumulated ^14^C_i_ to only 30 to 40% of the levels observed in D66 cells. These results indicate that BST1–3 play an important role in C_i_ uptake and fixation in low CO_2_ conditions in *Chlamydomonas*.

A bestrophin-like protein recently discovered in *Arabidopsis*, AtVCCN1, is a Cl^−^ channel that helps regulate the proton motive force (pmf) in the *Arabidopsis* thylakoid (17). Elimination of AtVCCN1 results in plants that have an increased pmf, altering how the plant regulates nonphotochemical quenching and the ΔpH across the thylakoid membrane. It is possible that the reduction of these BST proteins in *Chlamydomonas* could render cells less able to regulate the membrane potential (Δψ) and ΔpH components of the pmf, leading to photodamage or to an adenosine 5′-triphosphate (ATP)/NADPH imbalance. To investigate if *BST1*–*3*, in addition to being critical for C_i_ affinity and accumulation, have a role in regulating pmf similar to AtVCCN1, we measured electrochromic shift to estimate the pmf in the knockdown lines under HCO_3_^−^-depleted conditions (*SI Appendix*, Fig. S7). We found a small reduction of the pmf in the *bsti* mutants (*SI Appendix*, Fig. S7 *A* and *B*), which is opposite to what is seen in *Arabidopsis*. In addition, the pmf decayed slightly faster in the *bsti-1* and *bsti-3* mutants than in wild type (*SI Appendix*, Fig. S7*C*). We also measured the yield of variable chlorophyll *a* fluorescence to estimate photosystem II function in the mutants and found that F_v_/F_m_ was the same in mutant and wild-type cells (*SI Appendix*, Fig. S7*D*). The fact that the *bsti1*–*3* mutants grew normally at relatively high light levels (Fig. 4*A*) indicates that reducing *BST1*–*3* does not cause severe photodamage.

In conclusion, the localization, the C_i_ affinity, and the C_i_ accumulation phenotypes of the *bsti* triple-knockdown mutants support an essential role for *BST1*–*3* in the CCM.

## Discussion

We present evidence here that BST1–3 are chloroplast thylakoid localized anion transporters that are important components of the *Chlamydomonas* CCM. Cells that have reduced *BST1*–*3* transcript levels fail to grow on low CO_2_ (Fig. 4), have a lower affinity for C_i_ (Fig. 5), and have a reduced ability to accumulate added ^14^C_i_ (Fig. 6). A key aspect of current *Chlamydomonas* CCM models is that accumulated HCO_3_^−^ is converted to CO_2_ by CAH3, a carbonic anhydrase located in the thylakoid lumen (11–13). This feature of algal CCMs may extend to other algal types, notably diatoms, where Kikutani et al. (24) recently discovered a θ-type carbonic anhydrase within the thylakoid of *Phaeodactylum tricornutum* that was required for CCM function. These CCM models predict that a thylakoid HCO_3_^−^ transporter is required to deliver HCO_3_^−^ from the chloroplast stroma to the thylakoid lumen. We propose that BST1–3 are the transporters that bring HCO_3_^−^ to CAH3 inside the thylakoid.

Members of the human bestrophin family transport both HCO_3_^−^ and Cl^−^ ions (18). The homology modeling presented here supports the function of BST1–3 as anion transporters, with BST1–3 having predicted structural and conserved transport residue similarities to chicken and bacterial bestrophins (*SI Appendix*, Fig. S2).

The expression of all of the CCM transporters discovered previously is induced by ambient or lower CO_2_ conditions, and their expression is controlled by the transcription factor CIA5/CCM1 (22, 25). We have observed that all 3 *BST* genes are induced when *Chlamydomonas* is grown under ambient CO_2_ conditions (Fig. 2) and that this induction is absent in the *cia5* mutant (Fig. 2*A*). In addition, LCIB and LCIC, possible θ-carbonic anhydrases (24, 26) essential to the CCM (4) that interact with BST1–3 (15), have the same expression pattern (16). Thus, the expression of the *BST1*–*3* genes is consistent with these proteins playing a role in the uptake and accumulation of C_i_ when *Chlamydomonas* is exposed to low CO_2_ conditions.

An alternative hypothesis is that the 3 BST proteins have a function similar to AtVCCN1 (20) and are involved in Cl^−^ transport to regulate the pmf across the thylakoid. The presence of AtVCCN1 decreases pmf in *Arabidopsis*, but the presence of the 3 BST proteins increases pmf in *Chlamydomonas*. This result, in combination with our genetic and physiology data, suggests that the function of the BST proteins in *Chlamydomonas* is not the same as VCCN1 in *Arabidopsis*. A further understanding of this interconnection and the balancing/regulation of pmf within the context of the CCM is critical.

In *Chlamydomonas*, there seems to be a built-in redundancy of C_i_ transporter functions. For example, both LCI1 and HLA3 are present on the plasma membrane, and loss of only one of the proteins fails to cause an extreme growth phenotype at low CO_2_ (3). However, when more than 1 transporter is knocked down, a significant change in C_i_ uptake and growth is observed (4, 5). BST1–3 also appear to have redundant or overlapping functions. This is demonstrated in this study, as knocking out BST3 by itself did not cause a drastic change in growth or reduction in C_i_ affinity at ambient levels of CO_2_ (*SI Appendix*, Figs. S4 and S5). However, when the expression of all 3 genes is decreased in *bsti1*–*3,* cells could not grow at low CO_2_ and C_i_ uptake was severely compromised. In addition, *BST1*–*3* transcript levels in the RNAi strains correlated to C_i_ affinity and C_i_ uptake, supporting their C_i_ transport role and functional redundancy. This redundancy likely explains why *BST1*–*3* were not identified in earlier mutant screens, as these screens typically knock out only 1 gene at a time. The 3 BST proteins do, however, have sequence differences, particularly at their C termini. Therefore, they might have specific (or slightly different) physiological roles that cannot be differentiated under the conditions employed in this present study.

**Fig. 7.**
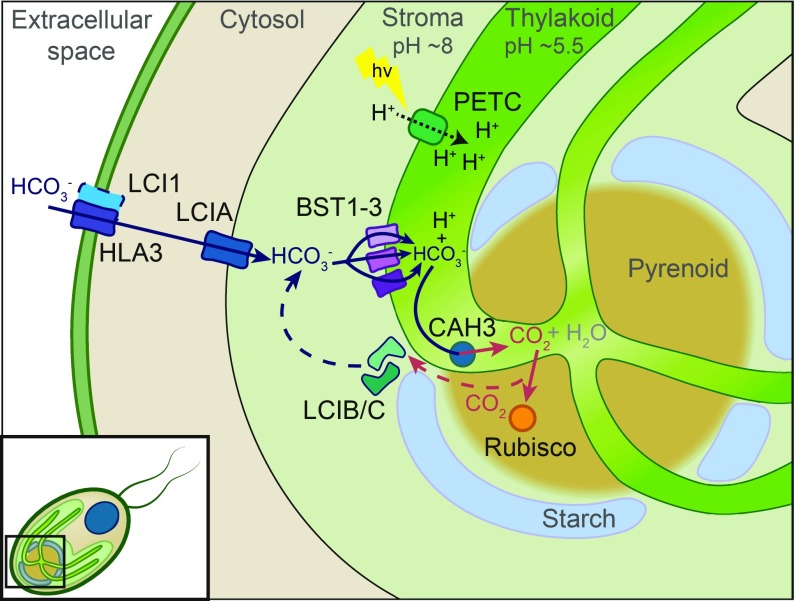
Tentative model showing the proposed physiological role of BST1–3 in the CCM of *Chlamydomonas*. Known transporters (LCI1, HLA3, and LCIA) are indicated on the plasma membrane and chloroplast, respectively. Solid line arrows indicate the movement of HCO_3_^−^ into the thylakoid by BST1–3. Dashed lines indicate the proposed leakage-reducing pathway that involves recycling CO_2_ by LCIB/C back to HCO_3_^−^. The dotted black line represents the light-driven establishment of a proton gradient across the thylakoid membrane by PSII and the cytochrome *b6f* complex of the photosynthetic electron transport chain (PETC).

Fig. 7 shows a refined model for the *Chlamydomonas* CCM, which now includes our proposed function for BST1–3. In this model, HLA3 and LCI1 transport HCO_3_^−^ across the plasma membrane, bringing HCO_3_^−^ into the cell (12, 13). At the chloroplast envelope, NAR1.2 (LCIA) transports HCO_3_^−^ into the chloroplast stroma. Then, BST1–3 on the thylakoid bring HCO_3_^−^ into the thylakoid lumen, where CAH3 in the pyrenoidal thylakoid tubules converts HCO_3_^−^ to CO_2_ to be fixed by Rubisco. Since BST1–3 are found throughout the thylakoid, a potential futile cycle is possible. However, for the futile cycle to take place, CAH3 needs to be present and active in the thylakoid membranes away from the pyrenoid. There is published work that CAH3 is preferentially located and activated in the pyrenoid tubules under low (<0.02%) CO_2_ conditions (8, 9). The location of BST1–3 is also likely to be important in the recapture of CO_2_ that is generated by the CCM (Fig. 7). Any CO_2_ in the pyrenoid not fixed by Rubisco has the potential to simply diffuse out of the cell (4, 27–29). The LCIB/C complex is thought to help recapture this CO_2_ (27) by directionally driving CO_2_ to HCO_3_^−^ or by acting as a tightly regulated carbonic anhydrase (30) at the pyrenoid periphery (28). This is interesting because there are data supporting the interaction of LCIB/C with BST1 and BST3 (15). Our model adds BST1–3 to this hypothesized recapture system (Fig. 7). Having BST1–3 throughout the thylakoid (Fig. 3) would increase the surface area for the reuptake of HCO_3_^−^ in the stroma. As such, we propose that the recapture of C_i_ is a 2-step process, with leaked CO_2_ from the pyrenoid converted to HCO_3_^−^ by LCIB/C and BST1–3 transporting the HCO_3_^−^ back into the thylakoid, creating an overall cyclic recapture mechanism. Loss of either BST1–3 or LCIB/C results in cells that cannot accumulate C_i_ to normal levels, which agrees with experimental observations.

The discovery of CCM components on the thylakoid (BST1–3) and inside the thylakoid lumen (CAH3) also indicates how light energy may be used to energize the CCM. The apparent pK_a_ of the interconversion of HCO_3_^−^ to CO_2_ is about 6.4. The pH of the chloroplast stroma, thought to be near 8.0, is well above the pK_a_, while the pH of the thylakoid lumen is thought to be close to 5.7 (14), below the pK_a_. When HCO_3_^−^ moves from the chloroplast stroma to the thylakoid lumen, it moves from an environment that favors HCO_3_^−^ to one that favors CO_2_. This effectively allows the algal cells to increase the CO_2_ concentration to levels higher than could be obtained by the action of carbonic anhydrase alone. Thus, a transthylakoid pH gradient is necessary for this proposed “CO_2_ pump,” and this pH gradient is set up by the photosystems and requires light. To date, all experimental data available indicate that light and the activity of the photosystems are required for the *Chlamydomonas* CCM to function. In fact, some of the earliest work in the field indicated that electron transport inhibitors and mutations that disrupt electron transport also inhibited the *Chlamydomonas* CCM (31, 32). One potential problem with this CO_2_ pump model is that it would partially reduce the pmf across the thylakoid membrane, thus reducing ATP biosynthesis. However, it should be pointed out that only a single H^+^ would be consumed per CO_2_ generated, which is the equivalent of less than one-third of an ATP per CO_2_ generated. This cost is far less than the 2 additional ATPs required for C_4_ photosynthesis, and C_4_ photosynthesis has been shown to be energetically competitive with C_3_ photosynthesis once the costs of photorespiration are considered (33). In conclusion, BST1–3 are bestrophin-like, thylakoid localized membrane proteins that are synthesized in coordination with other CCM components, and their predicted structures fit well with functionally characterized bestrophins. As such, they are excellent candidates to be the HCO_3_^−^ transporters that not only bring HCO_3_^−^ into the thylakoid lumen for CO_2_ generation but may also play a role in C_i_ recapture as well.

## Materials and Methods

### Cell Cultures, Growth, and Photosynthetic Assays.

*C. reinhardtii* culture conditions were set according to the conditions used previously (34). The D66 strain (*nit2*^−^, *cw15*, *mt*^+^) was obtained from Rogene Schnell (University of Arkansas, Fayetteville, AR), and CMJ030 (CC-4533; *cw15*, *mt*^−^) and *bst3* (*BST3* knockout LMJ.RY0402.089365) were obtained from the CLiP collection at the *Chlamydomonas* culture collection (23, 35). For acclimation experiments, Tris-acetate-phosphate–grown cells were switched to minimal media and bubbled with high CO_2_ (5% [vol/vol] CO_2_ in air) to reach an optical density at 730 nm between 0.2 and 0.3 (∼2 to 3 × 10^6^ cells per milliliter). This was followed by CCM induction when the cells were transferred to ambient CO_2_ (0.04% CO_2_) bubbling. For photosynthetic assays, cells acclimated to 5% or 0.04% CO_2_ were resuspended in C_i_-depleted buffer at pH 7.8 or pH 8.4, and O_2_ evolution was measured at different Ci concentrations. K_1/2_(C_i_) was calculated as the C_i_ concentration needed for the half-maximal rate of oxygen evolution.

### Fluorescence Protein Tagging and Confocal Microscopy.

The *BST1*–*3* genes driven by the constitutive *PSAD* promoter were cloned as reported by Mackinder et al. (15). Briefly, the open reading frames of *BST1*–*3* genes were PCR-amplified from genomic DNA and cloned into pLM005 with C-terminal Venus-3xFLAG and a *PSAD* promoter through Gibson assembly. *BST3* driven by its native promoter was cloned using recombineering based on methods reported by Sarov et al. (36). Transformation of these genes into *Chlamydomonas* and selection of colonies are described in *SI Appendix*, *SI Materials and Methods*. Images were captured with a laser-scanning microscope (LSM880; Zeiss) equipped with an Airyscan module using a 63× objective with a 1.4 numerical aperture. Argon lasers at 514 nm and 561 nm were used for excitation of Venus and chlorophyll, respectively. Filters were set at 525 to 550 nm for the Venus emission and at 620 to 670 nm for chlorophyll emission.

Additional details of materials and methods are provided in *SI Appendix*, *SI Materials and Methods*

## Supplementary Material

Supplementary File

## References

[1] Falkowski P., The global carbon cycle: A test of our knowledge of earth as a system. Science 290, 291–296 (2000). 11030643 10.1126/science.290.5490.291

[2] Bauwe H., Hagemann M., Fernie A. R., Photorespiration: Players, partners and origin. Trends Plant Sci. 15, 330–336 (2010). 20403720 10.1016/j.tplants.2010.03.006

[3] Yamano T., Sato E., Iguchi H., Fukuda Y., Fukuzawa H., Characterization of cooperative bicarbonate uptake into chloroplast stroma in the green alga *Chlamydomonas reinhardtii*. Proc. Natl. Acad. Sci. U.S.A. 112, 7315–7320 (2015). 26015566 10.1073/pnas.1501659112 PMC4466737

[4] Wang Y., Spalding M. H., Acclimation to very low CO_2_: Contribution of limiting CO_2_ inducible proteins, LCIB and LCIA, to inorganic carbon uptake in *Chlamydomonas reinhardtii*. Plant Physiol. 166, 2040–2050 (2014). 25336519 10.1104/pp.114.248294 PMC4256846

[5] Borkhsenious O. N., Mason C. B., Moroney J. V., The intracellular localization of ribulose-1,5-bisphosphate carboxylase/oxygenase in *chlamydomonas reinhardtii*. Plant Physiol. 116, 1585–1591 (1998). 9536077 10.1104/pp.116.4.1585 PMC35067

[6] Mackinder L. C., A repeat protein links Rubisco to form the eukaryotic carbon-concentrating organelle. Proc. Natl. Acad. Sci. U.S.A. 113, 5958–5963 (2016). 27166422 10.1073/pnas.1522866113 PMC4889370

[7] Rawat M., Henk M. C., Lavigne L. L., Moroney J. V., *Chlamydomonas reinhardtii* mutants without ribulose-1,5-bisphosphate carboxylase-oxygenase lack a detectable pyrenoid. Planta 198, 263–270 (1996).

[8] Blanco-Rivero A., Shutova T., Román M. J., Villarejo A., Martinez F., Phosphorylation controls the localization and activation of the lumenal carbonic anhydrase in *Chlamydomonas reinhardtii*. PLoS One 7, e49063 (2012). 23139834 10.1371/journal.pone.0049063 PMC3490910

[9] Mitra M., The carbonic anhydrase gene families of *Chlamydomonas reinhardtii*. Can. J. Bot. 83, 780–795 (2005).

[10] Moroney J. V., The carbonic anhydrase isoforms of *Chlamydomonas reinhardtii:* Intracellular location, expression, and physiological roles. Photosynth. Res. 109, 133–149 (2011). 21365258 10.1007/s11120-011-9635-3

[11] Karlsson J., A novel alpha-type carbonic anhydrase associated with the thylakoid membrane in *Chlamydomonas reinhardtii* is required for growth at ambient CO_2_. EMBO J. 17, 1208–1216 (1998). 9482718 10.1093/emboj/17.5.1208 PMC1170469

[12] Moroney J. V., Ynalvez R. A., Proposed carbon dioxide concentrating mechanism in *Chlamydomonas reinhardtii*. Eukaryot. Cell 6, 1251–1259 (2007). 17557885 10.1128/EC.00064-07 PMC1951128

[13] Spalding M. H., Microalgal carbon-dioxide-concentrating mechanisms: Chlamydomonas inorganic carbon transporters. J. Exp. Bot. 59, 1463–1473 (2008). 17597098 10.1093/jxb/erm128

[14] Takizawa K., Cruz J. A., Kanazawa A., Kramer D. M., The thylakoid proton motive force in vivo. Quantitative, non-invasive probes, energetics, and regulatory consequences of light-induced pmf. Biochim. Biophys. Acta 1767, 1233–1244 (2007). 17765199 10.1016/j.bbabio.2007.07.006

[15] Mackinder L. C. M., A spatial interactome reveals the protein organization of the algal CO_2_-concentrating mechanism. Cell 171, 133–147 e14 (2017). 28938113 10.1016/j.cell.2017.08.044 PMC5616186

[16] Fang W., Transcriptome-wide changes in *Chlamydomonas reinhardtii* gene expression regulated by carbon dioxide and the CO_2_-concentrating mechanism regulator CIA5/CCM1. Plant Cell 24, 1876–1893 (2012). 22634760 10.1105/tpc.112.097949 PMC3442575

[17] Herdean A., A voltage-dependent chloride channel fine-tunes photosynthesis in plants. Nat. Commun. 7, 11654 (2016). 27216227 10.1038/ncomms11654 PMC4890181

[18] Qu Z., Hartzell H. C., Bestrophin Cl^−^ channels are highly permeable to HCO_3_^−^. Am. J. Physiol. Cell Physiol. 294, C1371–C1377 (2008). 18400985 10.1152/ajpcell.00398.2007 PMC2465210

[19] Waterhouse A., SWISS-MODEL: Homology modelling of protein structures and complexes. Nucleic Acids Res. 46, W296–W303 (2018). 29788355 10.1093/nar/gky427 PMC6030848

[20] Qu Z., Chien L. T., Cui Y., Hartzell H. C., The anion-selective pore of the bestrophins, a family of chloride channels associated with retinal degeneration. J. Neurosci. 26, 5411–5419 (2006). 16707793 10.1523/JNEUROSCI.5500-05.2006 PMC6675304

[21] Kane Dickson V., Pedi L., Long S. B., Structure and insights into the function of a Ca^2+^-activated Cl^−^ channel. Nature 516, 213–218 (2014). 25337878 10.1038/nature13913 PMC4454446

[22] Fukuzawa H., Ccm1, a regulatory gene controlling the induction of a carbon-concentrating mechanism in *Chlamydomonas reinhardtii* by sensing CO_2_ availability. Proc. Natl. Acad. Sci. U.S.A. 98, 5347–5352 (2001). 11287669 10.1073/pnas.081593498 PMC33212

[23] Li X., An indexed, mapped mutant library enables reverse genetics studies of biological processes in *Chlamydomonas reinhardtii*. Plant Cell 28, 367–387 (2016). 26764374 10.1105/tpc.15.00465 PMC4790863

[24] Kikutani S., Thylakoid luminal θ-carbonic anhydrase critical for growth and photosynthesis in the marine diatom Phaeodactylum tricornutum. Proc. Natl. Acad. Sci. U.S.A. 113, 9828–9833 (2016). 27531955 10.1073/pnas.1603112113 PMC5024579

[25] Moroney J. V., Isolation and characterization of a mutant of *Chlamydomonas reinhardtii* deficient in the CO_2_ concentrating mechanism. Plant Physiol. 89, 897–903 (1989). 16666639 10.1104/pp.89.3.897 PMC1055941

[26] Jin S., Structural insights into the LCIB protein family reveals a new group of β-carbonic anhydrases. Proc. Natl. Acad. Sci. U.S.A. 113, 14716–14721 (2016). 27911826 10.1073/pnas.1616294113 PMC5187666

[27] Duanmu D., Wang Y., Spalding M. H., Thylakoid lumen carbonic anhydrase (CAH3) mutation suppresses air-Dier phenotype of LCIB mutant in *Chlamydomonas reinhardtii*. Plant Physiol. 149, 929–937 (2009). 19074623 10.1104/pp.108.132456 PMC2633820

[28] Yamano T., Light and low-CO_2_-dependent LCIB-LCIC complex localization in the chloroplast supports the carbon-concentrating mechanism in *Chlamydomonas reinhardtii*. Plant Cell Physiol. 51, 1453–1468 (2010). 20660228 10.1093/pcp/pcq105

[29] Kaplan A., Reinhold L., CO_2_ concentrating mechanisms in photosynthetic microorganisms. Annu. Rev. Plant Physiol. Plant Mol. Biol. 50, 539–570 (1999). 15012219 10.1146/annurev.arplant.50.1.539

[30] Mackinder L. C. M., The Chlamydomonas CO_2_ -concentrating mechanism and its potential for engineering photosynthesis in plants. New Phytol. 217, 54–61 (2018). 28833179 10.1111/nph.14749

[31] Badger M. R., Kaplan A., Berry J. A., Internal inorganic carbon pool of *Chlamydomonas reinhardtii:* Evidence for a carbon dioxide-concentrating mechanism. Plant Physiol. 66, 407–413 (1980). 16661446 10.1104/pp.66.3.407 PMC440644

[32] Spalding M. H., Spreitzer R. J., Ogren W. L., Carbonic anhydrase-deficient mutant of *Chlamydomonas reinhardii* requires elevated carbon dioxide concentration for photoautotrophic growth. Plant Physiol. 73, 268–272 (1983). 16663206 10.1104/pp.73.2.268 PMC1066451

[33] Moroney J. V., Jungnick N., Dimario R. J., Longstreth D. J., Photorespiration and carbon concentrating mechanisms: Two adaptations to high O_2_, low CO_2_ conditions. Photosynth. Res. 117, 121–131 (2013). 23771683 10.1007/s11120-013-9865-7

[34] Ma Y., Pollock S. V., Xiao Y., Cunnusamy K., Moroney J. V., Identification of a novel gene, CIA6, required for normal pyrenoid formation in *Chlamydomonas reinhardtii*. Plant Physiol. 156, 884–896 (2011). 21527423 10.1104/pp.111.173922 PMC3177283

[35] Zhang R., High-throughput genotyping of green algal mutants reveals random distribution of mutagenic insertion sites and endonucleolytic cleavage of transforming DNA. Plant Cell 26, 1398–1409 (2014). 24706510 10.1105/tpc.114.124099 PMC4036561

[36] Sarov M., A recombineering pipeline for functional genomics applied to Caenorhabditis elegans. Nat. Methods 3, 839–844 (2006). 16990816 10.1038/nmeth933

[37] Le S. Q., Gascuel O., An improved general amino acid replacement matrix. Mol. Biol. Evol. 25, 1307–1320 (2008). 18367465 10.1093/molbev/msn067

