# Supplementary material for: Thylakoid localized bestrophin-like proteins are essential for the CO2 concentrating mechanism of Chlamydomonas reinhardtii
Source: Proc Natl Acad Sci U S A. 2019 Aug 7;116(34):16915–20. doi: 10.1073/pnas.1909706116 (PMC6708349; doi:10.1073/pnas.1909706116)
Supplement: Supplementary File [file pnas.1909706116.sapp.pdf]

# Supplementary Information Appendix

## Thylakoid localized bestrophin-like proteins are essential for the $CO_2$ concentrating mechanism of *Chlamydomonas reinhardtii*.

Ananya Mukherjee[a], Chun Sing Lau[b], Charlotte E. Walker[b], Ashwani K. Rai[a], Camille I. Prejean[a], Gary Yates[b], Thomas Emrich-Mills[b], Spencer G. Lemoine[a], David J. Vinyard[a], Luke C.M. Mackinder[b1], and James V. Moroney[a1]

[1]Correspondence:
btmoro@lsu.edu (JV Moroney)
luke.mackinder@york.ac.uk (LCM Mackinder

**This PDF file includes:**

Supplementary Materials and Methods
Supplemental Figures S1 to S7
Table S1
References for SI reference citations

## SI Materials and Methods

### Cell cultures, growth and photosynthetic assays

*Chlamydomonas reinhardtii* culture conditions were set according to the conditions used previously (1). The D66 strain (*nit2*$^-$, *cw15*, *mt*$^+$) was obtained from Dr. Rogene Schnell (University of Arkansas, Little Rock) and CMJ030 (CC-4533; *cw15*, *mt*$^-$) and *bst3* (BST3 knockout LMJ.RY0402.089365) were obtained from the CLiP collection at the *Chlamydomonas* culture collection (2, 3). Tris-Acetate-Phosphate (TAP) and Minimal (MIN) media (acetate free) were prepared according to Sueoka (4). TAP and MIN plates for growth were made by adding 1.2% (w/v) agar. Colonies from TAP plates were used to inoculate 100 mL TAP liquid for mixotrophic growth. Growth in liquid TAP was under continuous illumination (100 µmol photons



www.pnas.org/cgi/doi/10.1073/pnas.1909706116

$m^{-2}$ $s^{-1}$) and shaking for 48 h. Early log phase TAP cultures were harvested and washed with MIN media, followed by resuspension MIN media and bubbled with high $CO_2$ (5% [v/v] $CO_2$ in air) to reach $OD_{730}$ between 0.2 and 0.3 (~2-3 x$10^6$ cells $mL^{-1}$). This was followed by CCM induction when the cells were transferred to ambient $CO_2$ (0.04% $CO_2$) bubbling. For photosynthetic assays, cells acclimated to 5% or 0.04% $CO_2$ were resuspended in $C_i$-depleted buffer at pH 7.8 or 8.4 and $O_2$ evolution was measured at different $C_i$ concentrations.

**Photosynthetic assays**

100 mL TAP was inoculated with colonies of *Chlamydomonas* cultures and grown for 48 h in the light (100 µmol photons $m^{-2}$ $s^{-1}$) to logarithmic phase. Cells were then washed with MIN media and transferred to MIN media that was bubbled with high $CO_2$ until they reached a cell density $OD_{730}$ = 0.2 - 0.3. The cells were then bubbled with ambient $CO_2$ in the light for 12 h to induce the CCM. The external $C_i$ was estimated according to Ma et al. (1). Briefly, cells equivalent to 100 µg chlorophyll were suspended in HEPES-NaOH buffer (pH 7.4) or 25 mM EPPS-NaOH buffer (pH 8.4) that had been bubbled with nitrogen gas. An $O_2$ electrode chamber (Rank Brothers, Cambridge UK) illuminated at 300 µmol photons $m^{-2}$ $s^{-1}$, was used to deplete $C_i$ until no net oxygen exchange is seen. Increasing concentration of $NaHCO_3$ was then injected into the depleted cells. $K_{1/2}(C_i)$ was calculated as the DIC concentration needed for half maximal rate of oxygen evolution.

**Inorganic carbon uptake**

Silicone oil centrifugation was used to measure intercellular concentration of dissolved $C_i$ as in Moroney et al. (5). Briefly, cells were centrifuged and suspended at 25 µg chlorophyll (Chl) $mL^{-1}$ density in $C_i$ depleted 25 mM EPPS-NaOH (pH 7.8 or 8.4) and incubated in the light until net $O_2$ evolution was zero. Cells were maintained in the light until used. 300 µL of $C_i$ depleted cells were then centrifuged in tubes containing 25 µL of 1 M glycine (pH 10) with 0.75% (w/v) SDS overlaid with 75 µL of Dow Corning AR 20 silicone oil. Assays were performed at 25 °C in 200 µmol photons $m^{-2}$ $s^{-1}$ light in a Beckman Microfuge B. $C_i$ uptake was initiated by adding either 3 µl of 25 mM (pH 7.8) or 50 mM (pH 8.4) $NaH^{14}CO_3$ followed by the indicated time of illumination (between 15 and 120 sec at 150 µmol photons $m^{-2}$ $s^{-1}$). The reaction was terminated by a 15 sec centrifugation in a Microfuge B (Beckman). Internal $C_i$ was calculated using the difference between total and acid stable $^{14}C$ in the pellet and corrected for cell volume (6).

## Generation of RNAi constructs

Artificial microRNA constructs for the knock-down of the BST proteins were made using the protocol of Molnar et al. (7). The Web MicroRNA Designer (WMD3) website (http://wmd3.weigelworld.org/cgi-bin/webapp.cgi) was used to find target sequences that align to the "common region" of *BST1-3* coding sequences. The Designer page of WMD3 was used to design oligos from the target regions selected. Annealed oligos were then ligated to the unique *SpeI* digested site of pChlamyRNA3int plasmid, obtained from the *Chlamydomonas* resource center. Table S1 has the list of oligos used for the miRNA constructs, under the region miRNA, with the target sequences underlined.

## Mutant isolation and phenotypic screen

Assembled miRNA constructs were transformed into the wild type, D66, by electroporation (8). Transformants were selected on TAP agar media containing the antibiotic paromomycin (4 µg $mL^{-1}$; Invitrogen) and maintained in high $CO_2$ (5% $CO_2$ in air). A 1000 paromomycin resistant strains were then replica plated on MIN media plates and grown in a high $CO_2$ chamber and a low $CO_2$ chamber (<0.02% $CO_2$) with continuous illumination (100 µmol photons $m^{-2}$ $s^{-1}$) for 7 days. Colonies were screened for "sick on low $CO_2$" phenotype as compared to D66 and *cia3*. Selected colonies were grown in TAP to log phase and then resuspended in MIN media to 6.6 x $10^5$ cells $mL^{-1}$ followed by serial dilution of 1:10 three times. Spot tests were done by spotting 15 µl of each sample on MIN plates. These plates were placed in high (5% $CO_2$ in air), ambient (~0.04% $CO_2$) and low $CO_2$ (<0.02% $CO_2$) chambers under continuous illumination at 100 µmol photons $m^{-2}$ $s^{-1}$ for 7 days. The $CO_2$ concentration was measured using an Environmental Gas Monitor (EGM-4, PP systems, Massachusetts).

## Confirmation of flanking region

Primers specific to the *BST3* gene were made (complementary to the last exon) to span the insert in the *bst3* mutant. Insert specific primers and the information for the flanking region were

obtained from the CLiP website (https://www.chlamylibrary.org/allMutants) (3). The primers are shown in Table S1 under the section *bst3* primers.

**Gene expression analysis**

RNA was extracted using Trizol reagent following the method provided by Invitrogen. 1 µg RNA per sample was used as template for cDNA, which was made using ProtoScript® First Strand cDNA Synthesis Kit (NEB) as per manufacturer's instructions. 100 ng RNA per sample was used to conduct qRT-PCR using the Luna® Universal One-Step RT-qPCR Kit from NEB as per manufacturer's instructions using QuantStudio 6. Actin primers were used for semi quantitative RT-PCR and CBLP was used as a reference gene for all qRT-PCR. All primers used are listed in Table S1.

**Protein isolation and immunoblot analysis**

All cell cultures for BST3 protein quantification were grown in MIN media and subjected to high $CO_2$ for 48 h to promote growth and then transferred to ambient $CO_2$ for 48 h to initiate the CCM. Cells were harvested by centrifugation once they reached mid-exponential growth phase (2-4 x $10^6$ cell $mL^{-1}$) and were resuspended in 3 mM HEPES-KOH, pH 7.5, 60 mM dithiothreitol, 60 mM $Na_2CO_3$, 12 % sucrose, 2 % SDS and protease inhibitors for total protein isolation (9). Samples were subsequently boiled for 50 sec, flash frozen in liquid $N_2$ and stored at -80 ºC prior to analysis. Samples were normalized to Chl content (2.5 µg Chl in total) and resolved by SDS-PAGE on 10 % polyacrylamide gels (Mini-PROTEAN TGX, Bio-Rad Laboratories). Proteins were transferred to a PVDF-FL membrane on a Bio-Rad semidry blotting system. The membranes were blocked in 5% milk TBST (TBS containing 0.1% Tween) for 2 - 3 h at room temperature. BST3 primary antibody (rabbit) was generated against the amino acid sequence CSHSNGNGSKPVSTQVP-amide (Yenzym). Alpha-tubulin primary antibody (mouse) was acquired from Agrisera. Primary antibodies (both 1:1000 dilutions) were applied in a cocktail in 3 % milk TBST and incubated at 4 ºC overnight. Anti-rabbit and anti-mouse fluorescently tagged secondary antibodies, Invitrogen AlexaFluor 488 and 555, respectively, were applied to PVDF-FL membranes diluted in 3% milk TBST at a 1:20 000 dilution for 1 h at

room temperature and protected from light. Immunoblots were imaged using an Amersham Typhoon 5 Scanner with 488 and 535 excitation lasers and the Cy2 and Cy3 emission filters.

**Image analysis**

Quantification of BST3 and alpha-tubulin fluorescence from the scanned immunoblots was performed using ImageQuant TL software (GE Healthcare Life Sciences) with the Analysis ToolBox package. Prior to the BST3 quantification, the fluorescence background signal was removed and all BST3 measurements were normalized to alpha-tubulin loading control. Quantification of BST3 localization under native promotor was performed in Fiji (Image J v2.0.0) (10). Regions of interest (ROI) were defined (chloroplast and pyrenoid periphery) for each individual cell using the chlorophyll channel. Venus fluorescence was quantified as a parameter of BST3 abundance using the pre-defined ROIs. BST3 enrichment in the pyrenoid periphery is presented as a ratio of pyrenoid periphery to chloroplast Integrated Density.

**In silico modelling of bestrophin-like proteins**

Peptide sequences of BST1-3 were obtained from Phytozome v12.1. Homology modelling of BST1-3 was achieved using Swiss-model webserver using *Klebsiella pneumoniae* bestrophin (PDB: 4DW8) as a template. Model structures were visualized with Chimera 1.13.1 (11). The obtained BST1 homopentamer model was submitted to energy minimization with GROMOS 43B1 forcefield and the electrostatic potential was calculated using atom partial charge using Swiss-PDBviewer (V4.01). Result are visualized on the molecular surface. BST1 homopentamer structure quality was obtained using the QMEAN score (12, 13).

**Phylogenetic tree**

Amino acid sequences for BST1 (Cre16.g662600.t1.2), 2 (Cre16.g663400.t2.1) and 3 (Cre16.g663450.t1.2) were BLASTED against NCBI Genbank (14) and Phytozome v12.1 (15) and the top hits downloaded. Additionally, amino acid sequences encoding *Homo sapiens* BEST1 (SJM31533.1) and *K. pneumoniae* bestrophin (pdb_4WD8_A) were downloaded from NCBI Genbank to be included as the outgroup in the phylogenetic analysis. A total of 63 initial sequences were aligned in Geneious 11.1.4 (16) using a ClustalW (17) algorithm with the amino acid substitution matrix BLOSUM62 (18). Duplicate sequences from the two databases (NCBI

and Phytozome) and sequences with a pairwise percentage positive identity (BLOSUM62) of less than 70% were removed. The final alignment included 30 sequences and was manually trimmed to remove variable length ends from the sequences. The phylogenetic analysis was completed in MEGA X (19). The best Maximum Likelihood (ML) model for phylogenetic analysis of the alignment was calculated using the Model Selection function in MEGA X. A ML tree was constructed using the LG substitution model (20) with Gamma distribution (5 discrete categories) and 500 bootstrap replicates. Adobe Illustrator CC was used to prepare the tree for publication.

**Chlorophyll fluorescence and electrochromic shift**

Cell cultures in exponential phase were diluted to 2 µg Chl $mL^{-1}$ in MIN media. 5 mM $NaHCO_3$ was added and 3 mL samples were dark adapted for 2 min. $F_o$ and $F_m$ were measured using a commercial PAM fluorometer (FL 3000, Photon Systems Instruments, Brno, Czech Republic). $F_v/F_m$ was calculated as $(F_m – F_o)/F_o$. Cell cultures were briefly centrifuged and resuspended in minimal media to OD = 4 at 730 nm. The $C_i$ of cells was depleted in 25 mM EPPS-NaOH buffer (pH 8.4) using an $O_2$ electrode chamber (Rank Brothers, Cambridge UK) illuminated at 300 µmol photons $m^{-2}$ $s^{-1}$ until no net oxygen exchange is seen. Then 3 mL samples were transferred to a fluorescence cuvette and measured using a MultispeQ v1.0 spectrometer (PhotosynQ, East Lansing, MI) with the “Chlamy MultispeQ v1.0 beta” protocol (21). Actinic light intensity used to generate pmf was 400 µmol photons $m^{-2}$ $s^{-1}$. Raw data from the ECS measurement were converted to absorbance units, normalized, and fit to a one-component exponential decay function using OriginPro.

```
BEST1        ---------------------------------------------------------MTI   3
AtVCCN1      MYQSMNLSVSSNFTHR--SLLESRFPI-----FSTGFRKSVNLKPPRVSSGPESNDSGHE   53
BST1         MQMQANRSSLRASPVRGLGARPLLRALPAGRVARLNV--SAQAKDPNAPI--QSNPLGT-   55
BST2         MQCLSSRPVA--MGRAGSSALPR-LPLRAGRVCHLGVRCQAANKDPNAPI--QSNPLGS-   54
BST3         MQ----VSKV--PSSASARCLPR-LPVRTSRVCQLSVRCQAANKDPNAPI--QSNPLGS-   50


BEST1        TYTSQVANARLGSFSRLLLCWRGSI----------------------YK----------   30
AtVCCN1      TLTDKL-----IHLLRAVPDWADEIKERGMQQKRSLYTHEKWVEHRSSLRHVRHLLSSFS   108
BST1         -LSSQ---------SGQVAT--LPRSEEARKYFRTVYDFPQWQKHRSSYRFAERLFQLSQ   103
BST2         -FSSQL-----Q--NQ--PT--LPRSEEARKYFRTVYDFPQWQTHRNQYRLMKRLFSIPQ   102
BST3         -FSSQN-----S--SGAVVT--APRNEDARKYFRTVYDFPQWQKHRSQSRLVRRLFTIPQ   100
               :.:                                            :

BEST1        ------LLYGEFLIFLLCYYIIRFIYRLALTEEQQ-LMFEKLTLYCDSYIQLIPI--SFV   81
AtVCCN1      SRVILSLIPPVFFFTSVAVVIASYNSAVALDWLPGIFPILRSSSL---PYQLTAPALALL   165
BST1         SHILQNALPAISWVTLVATLVASYGYSYDQHMLPDVFPSISPNASCTAFISNTSVALSLL   163
BST2         SHVIQNALPSIMWVAFTSTCVAAYMYGYDQHMLPEGFPTLAPNAACSAFISNTSVALSLL   162
BST3         SHVIQNALPSIMWVTFTSTCVAAYMYGYDLHILPEGFPTLAPNAACSAFISNTSVALSLL   160
                    :     .   .  :  :            :     .       .      :::

BEST1        LGFYVTLVVTRWWNQYENLPWPDRLMSLVSGFVEGKDEQGRLLRRTLIRYANLGNVLILR   141
AtVCCN1      LVFRTEASYSRYEEG--RKAWV--------GIIAGTND---LARQVICSVDSSGDELIIK   212
BST1         LVFRTNSSYGRWDEA--RKMWG--------GLLNRSRD---IMRQGATCF--PDDQVEAK   208
BST2         LVFRTNSSYGRWDEA--RKMWG--------GLLNRSRD---IMRQGATCF--PDDQVEAK   207
BST3         LVFRTNSSYGRWDEA--RKMWG--------GLLNRSRD---IMRQGATCF--PDDQVEAK   205
             * * .     *: :   .  *         *::  . :   : *:        .: :  :

BEST1        SVSTAVYKRFPSAQHL-V---------QAGFMTPAEHKQLEKLSLPHNMFWVPWVWFANL   191
AtVCCN1      DLLLRYIAAFPVALKCHVIYGSDIARDLRNLIEADDLSLILQAK--H----RPRCVIEFI   266
BST1         KALARWTVAFSRALRIHFQPEVTIESELQNILTPAELQMLAKSQ--H----RPVRAIHAI   262
BST2         KALARWVVAFSRALRIHFQPEVTIESELKNILTPAELQMLAKSQ--H----RPVRAIHAI   261
BST3         KALARWTVAFARALRIHFQPEVTIESELQNILTPAELQMLAKSQ--H----RPVRAIHAI   259
             .        *  * :  .           .::   : . : : .  *     *   :  :

BEST1        SMKAWLGGRIRDPILLQSLLNEMNTLRTQCGHLYAYDWISIPLVYTQVVTVAVYSFFLTC   251
AtVCCN1      S--QSIQLLKLDDAKRDLLESKMLHLHEGIGVCEQLMGIPIPLSYTRLTSRFLVFWHL--   322
BST1         S--QIIQSVPMSSIHQQQMSNNLTFFHDVLGGCERLLRAPIPVSYTRHTARFLFAWLT--   318
BST2         S--QIIQSVPMSSIHQQQMSNNLTFFHDVLGGCERLLRAPIPVSYTRHTARFLFAWLT--   317
BST3         S--QIIQSVRMSSIHQQQMSNNLTFFHDVLGGCERLLRAPIPVSYTRHTARFLFAWLT--   315
             *    :     .    : : .::  ::   *         **: **: .:  :  :

BEST1        LVGRQFLNPAKAYPGHELDLVVPVFTFLQFFFYVGWLKVAEQLINPFGEDDDDFETNWIV   311
AtVCCN1      ------TLPIILWDECHWI-VVPATFIS-AASLFCIEEVGVLIEEPFPMLALDELCDLVH   374
BST1         ------LLPFALYPTTGWG-VVPVCTGI-AAVLCGIEEIGVQCEEPFGILPLDVICNRIQ   370
BST2         ------LLPFALYGSCGVS-VIPVCSGI-AAVLCGIEEIGVQCEEPFGILPLDVICNRIQ   369
BST3         ------LLPFALYGSCGVS-VIPVCTGI-AAVLCGIEEIGVQCEEPFGILPLDVICNRIQ   367
                     *   :       *:*.             ::.    :**     *   : :

BEST1        D---------RNLQVSLLAVDEMHQDLPRMEPDMYWNKPEPQPPYTAASAQFRRASFMGS   362
AtVCCN1      SNIQEAVKSEKVIRNRIIAKIKLHEFKHSSNGRH---RS--------------------   410
BST1         ADVMATLKDDADTKT-ILAEAGLISLIPSATSATPVASAEPVLVSARPSAAP--APN---   424
BST2         ADVMATLKDDADTKT-ILAEAGLISLRANSAMAVENALPDLDSINAAA------PNG---   419
BST3         ADVMATLKDDADTKT-VLAEAGLISLIPSMSLPPTEHASPSDPVTAAAAAALAAANG---   423
                          :  ::*   : .
```

**Fig. S1.** Multiple sequence alignment of BST1-3, BEST1 (human) and AtVCCN1 (an Arabidopsis voltage gated chloride channel). CLUSTAL Omega was used for the multiple sequence alignment and TMHMM for predicting the TM domains (in yellow) in BSTs of *Chlamydomonas*. Overall there is an average of 80% similarity within BST1-3, ~30% between BST1-3 and AtVCCN1 and 21 – 23 % between BST1-3 and BEST1. * indicates conserved amino acids

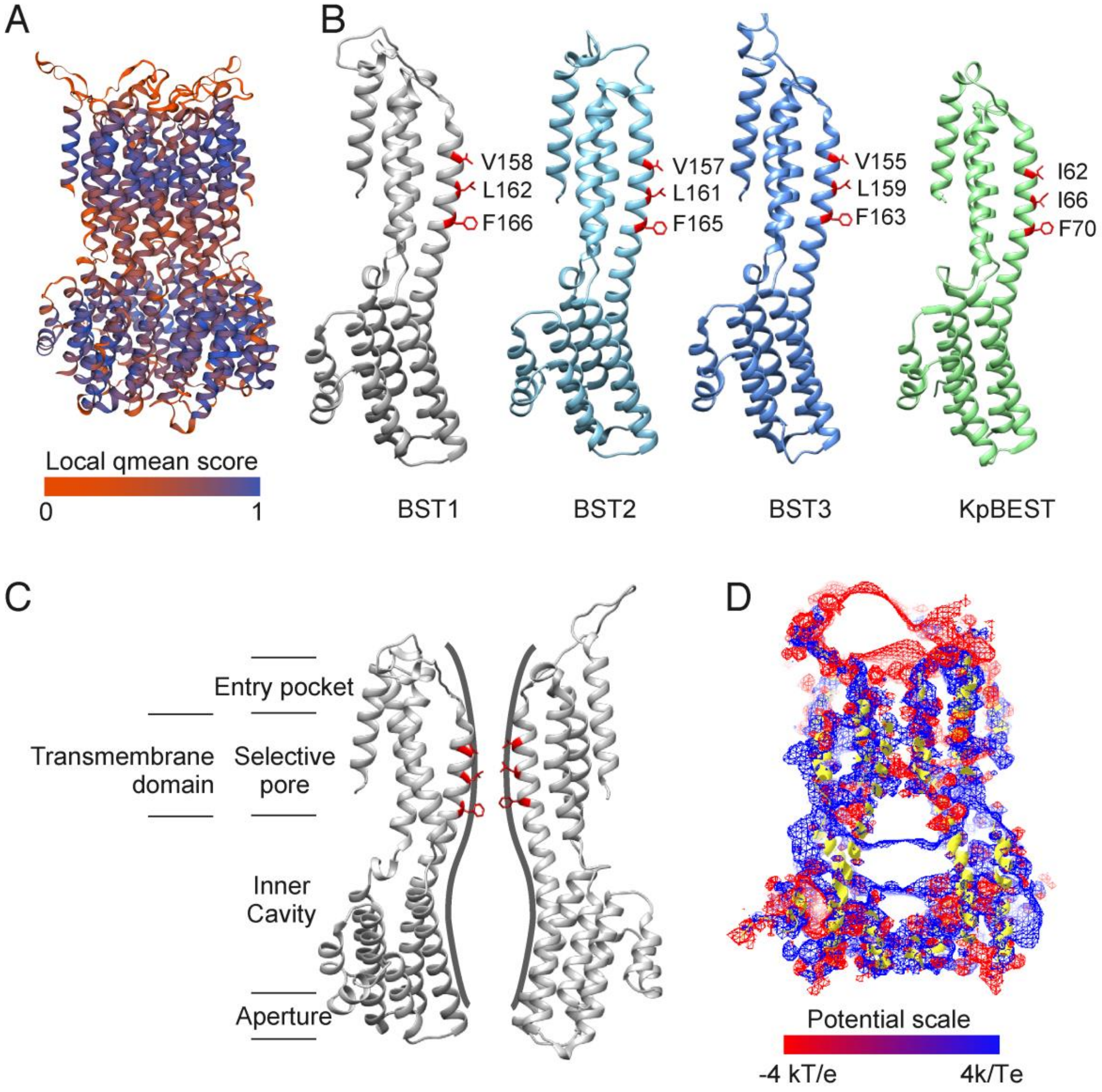


**Fig. S2.** Structural models for BST1-3. (A) Quaternary structure of BST1 pentamer model colored with the local QMEAN score - a structure quality assessment scoring function. (B) Structural models for *Chlamydomonas* BST1-3 and *Klebsiella pneumoniae* bestrophin (kpBEST). BST1-3 structural models were obtained using Swiss-model server with the kpBEST crystal structure (PDB: 4DW8) as template. The obtained structures and that of kpBEST are displayed as monomers with conserved residues lining the selective pore highlighted in red. (C) Outline of the channel cavity is drawn on BST1 homopentamer, with only two subunits shown for clarity. (D) Calculated electrostatic potential (red/ blue) is shown on BST1 model (yellow).

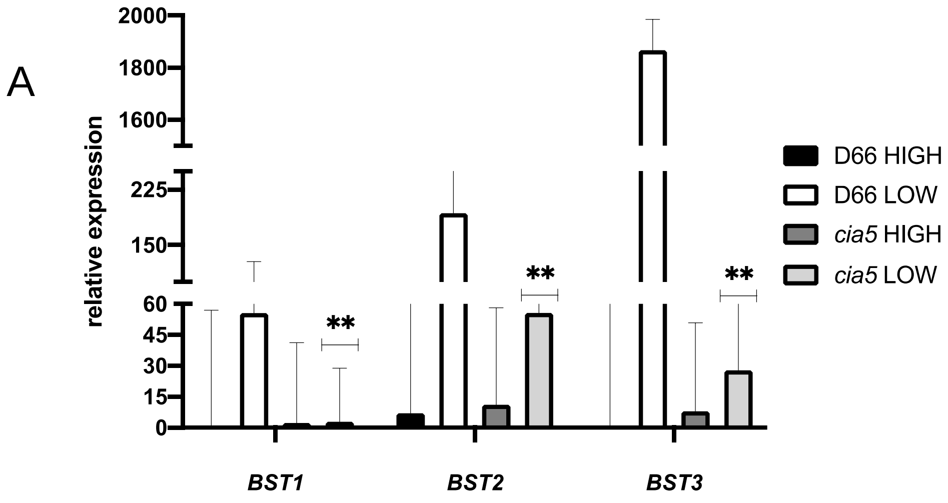


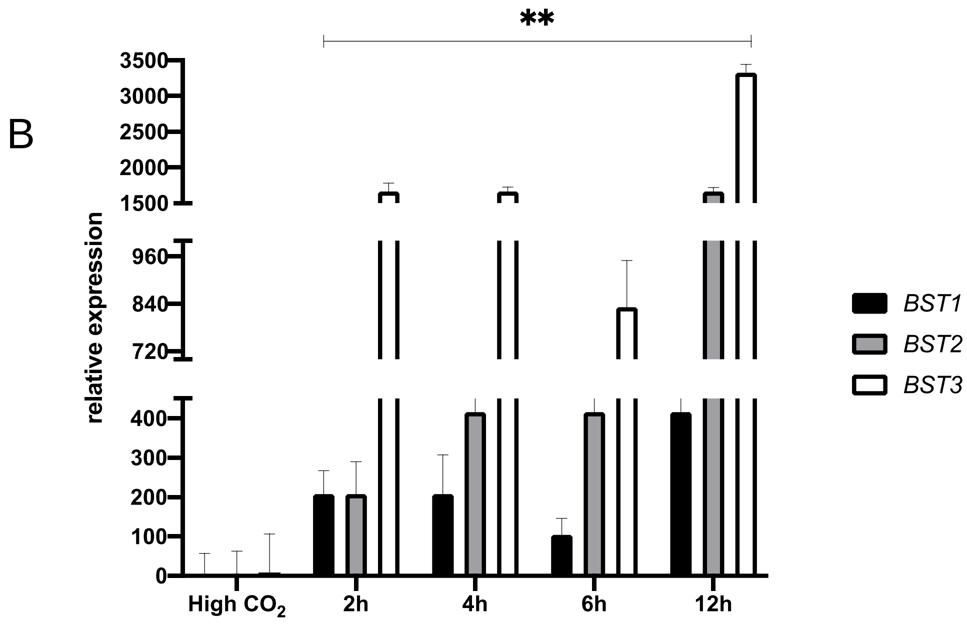


**Fig. S3** Transcript analysis of *BST1-3*. (A) qRT-PCR showing *BST1-3* accumulation in ambient $CO_2$ (0.04 % $CO_2$) vs. high $CO_2$ (5 % (v/v) $CO_2$ in air) in D66 and *cia5* cells. Data relative to *BST1* D66 high $CO_2$. ** shows that *cia5* low $CO_2$ expression is significantly lower than D66 low $CO_2$ expression (One-tailed Student's T-Test $p < 0.01$) (B) qRT-PCR time course showing the expression of *BST1-3* in cDNA obtained from high $CO_2$ (5 % $CO_2$ (v/v) in air) and in cells switched to ambient $CO_2$ (0.04 % $CO_2$) for the indicated times. Data plotted as relative expression to high $CO_2$. ** show significant upregulation relative to high $CO_2$ (One-tailed Student's T-Test $p < 0.01$). All values have been normalized to CBLP.

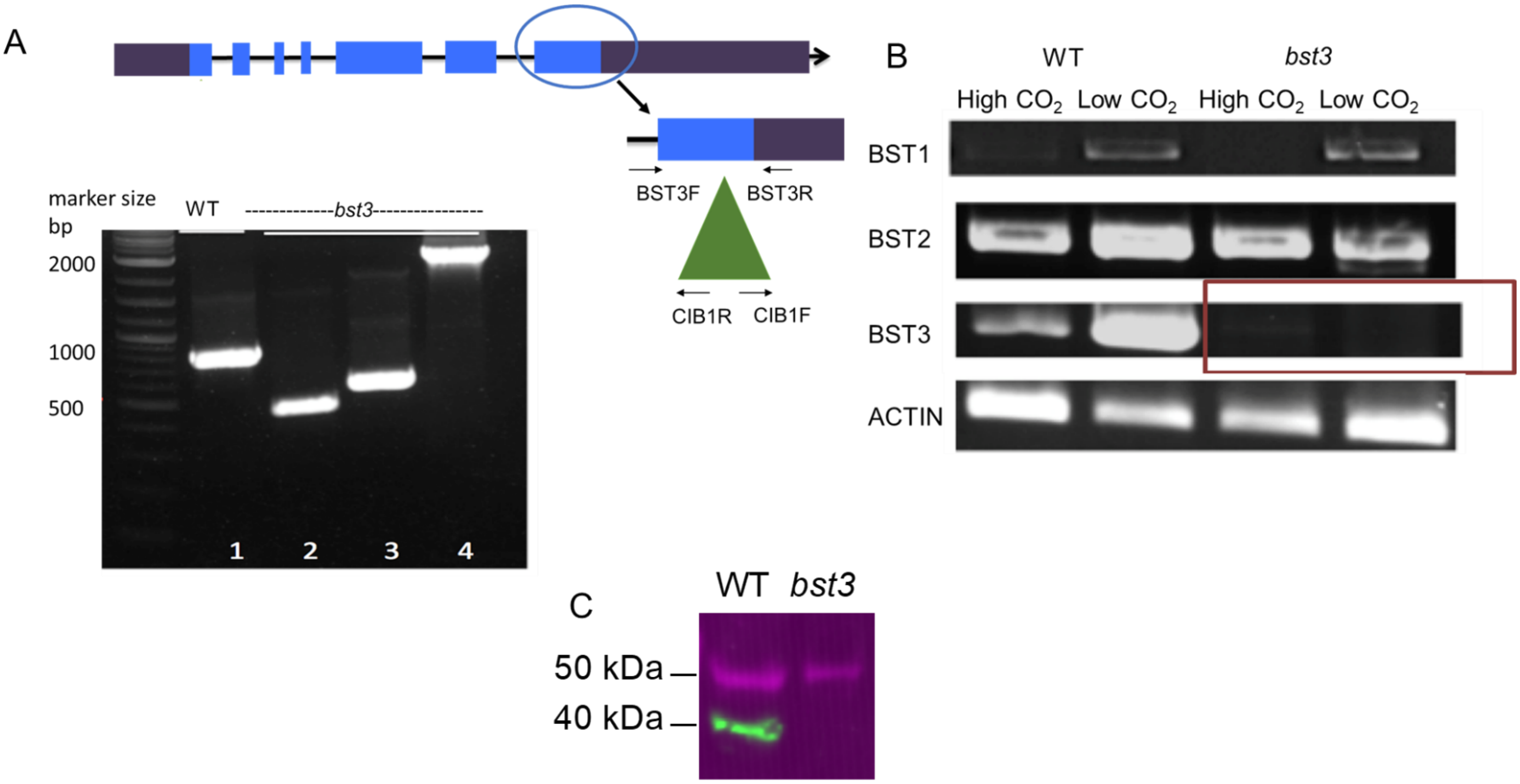


**Fig. S4** BST3 is knocked out in the CLiP mutant *bst3*. (A) The position of the insert in the BST3 gene and the PCR reactions used in the confirmation of the insert in *bst3*. The insert position was confirmed with primers specific for the insert (CIB1F/R) and to the gene (BST3F/R). Lane 1, BST3F and BST3R primers using CLiP-WT DNA as template (expected size 1kb); lane 2, CIB1R (insert) and BST3F primers using *bst3* DNA as template (expected size 0.6 kb); lane 3, CIB1F (insert) and BST3R primers using *bst3* DNA as template (expected size 0.8 kb); lane 4, BST3F and BST3R primers using *bst3* DNA as template (expected size 3 kb). The size difference between lane 1 and 4 shows that there is an 1800 bp cassette. (B) Semi quantitative RT-PCR showing BST1-3 accumulation in ambient $CO_2$ vs. high $CO_2$ in WT and *bst3* cells. Actin is used as a loading control. (C) Western blot of BST3 (green) shows the absence of BST3 in the *bst3* mutant. α-tubulin loading control is also pictured (magenta).

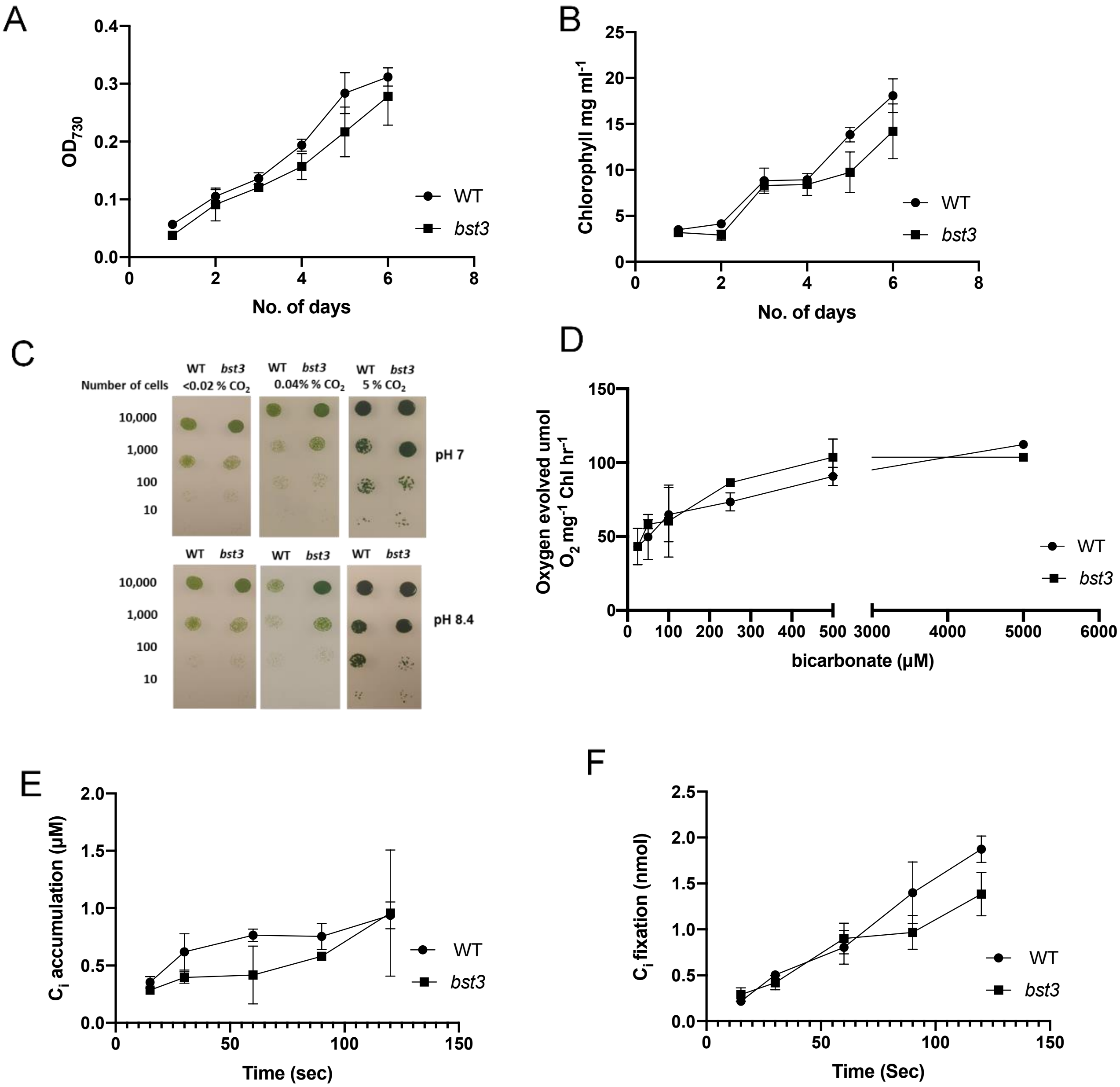


**Fig. S5** Growth and inorganic carbon affinity of *bst3*. (A) Growth of WT vs. *bst3* at pH 8.6 was measured using $OD_{730}$ and (B) by chlorophyll estimation at wavelength 645 and 663 nm. Growth was at ambient $CO_2$ for six days. Cells were grown in TAP for 48 hours before transferring them to MIN at an $OD_{730}$ of 0.01. (C) Spot test showing the growth of *bst3* and WT in low $CO_2$, ambient $CO_2$ and high $CO_2$. Cells were diluted to 6.6 x $10^5$ cells $mL^{-1}$ followed by serial dilution of 1:10 three times. (D) Oxygen evolving activity was measured at pH 8.4 and the $C_i$ concentration needed for half maximal rates of oxygen evolution ($K_{1/2}(C_i)$) were calculated from the $O_2$ evolution versus $C_i$ curves. Triplicate runs were made at each $C_i$ concentration. (E) and (F) Inorganic carbon uptake of WT and *bst3*. $C_i$ uptake and fixation was estimated in WT and *bst3* using the silicone oil uptake method (see Materials and Methods). Cells were grown in TAP media and then acclimated to ambient $CO_2$ in MIN media for 12 h prior to the assays. Cells were harvested and depleted of endogenous $C_i$ before running the assays. A time course of intracellular $C_i$ uptake (E) and fixation (F) at pH 8.4 are shown. Triplicate samples were run for each time point. The added $H^{14}CO_3^-$ concentration was 25 µM at pH 8.4.

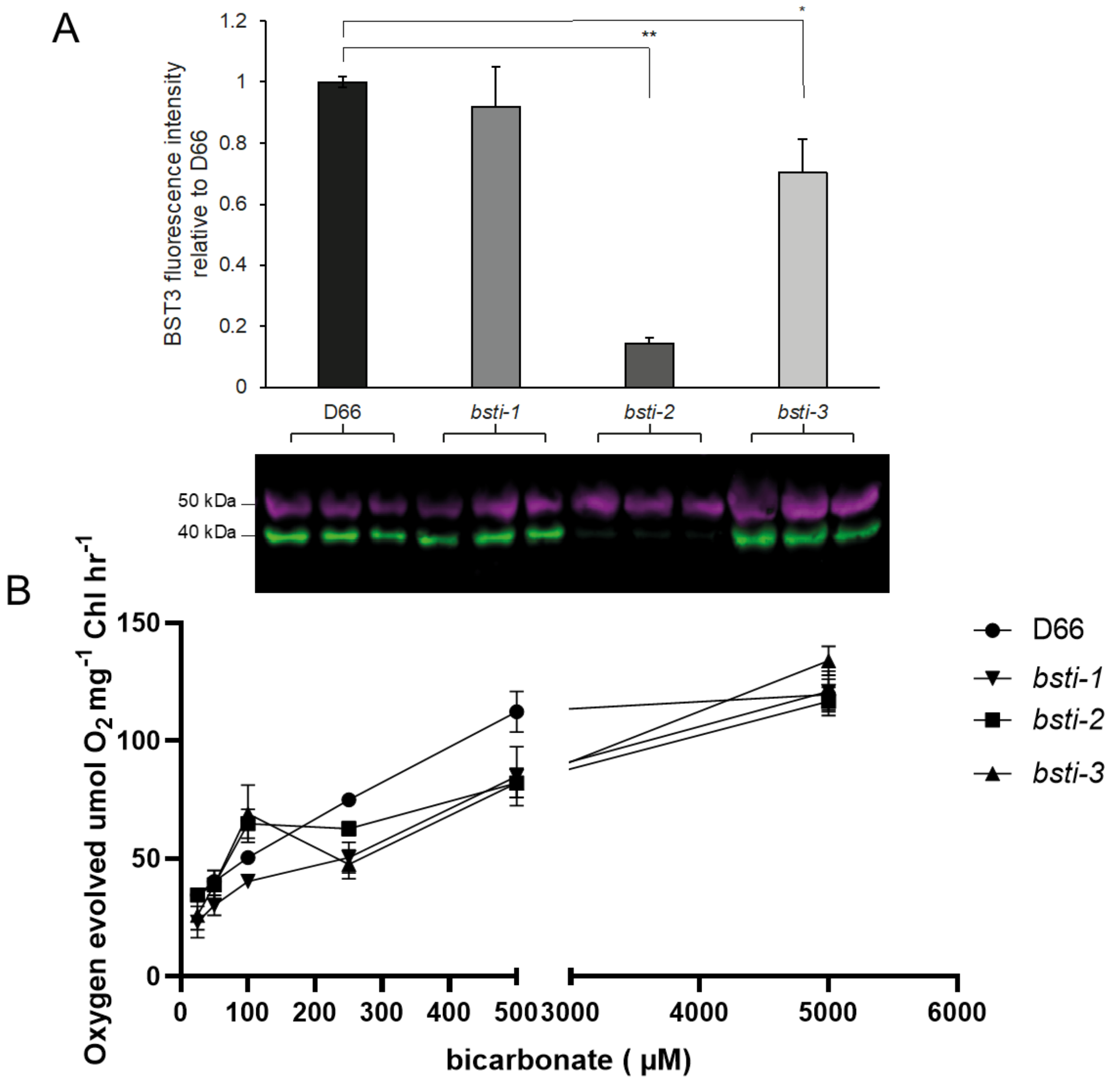


**Fig. S6** Immunoblot and high $CO_2$ acclimated photosynthetic oxygen evolution activity of *bsti1-3* and D66. (A) Immunoblot using fluorescent secondary antibodies shows BST3 (green) and α-tubulin (magenta) protein levels in D66 wild-type and *bsti1-3* RNAi lines. Fluorescent intensity of BST3 normalized to the α-tubulin loading control and presented relative to D66 as a measure of protein production. RNAi lines *bsti-2* and *bsti-3* show a significant reduction in BST3 compared to D66 when analysed using a One-tailed Students T-Test, $p < 0.01$ (**) and $p < 0.05$ (*) respectively. Error bars denote standard error. (B) $C_i$ affinity was determined for *bsti1-3* and D66 acclimated to high $CO_2$ (5% $CO_2$) for 12 h at pH 7.8. Oxygen evolving activity was measured and the $K_{1/2}(C_i)$ values ($C_i$ concentration needed for half maximum oxygen evolution) were calculated from the $O_2$ evolution versus $C_i$ curves. Triplicate runs were done at each $C_i$ concentration. Error bars are all based on standard deviation.

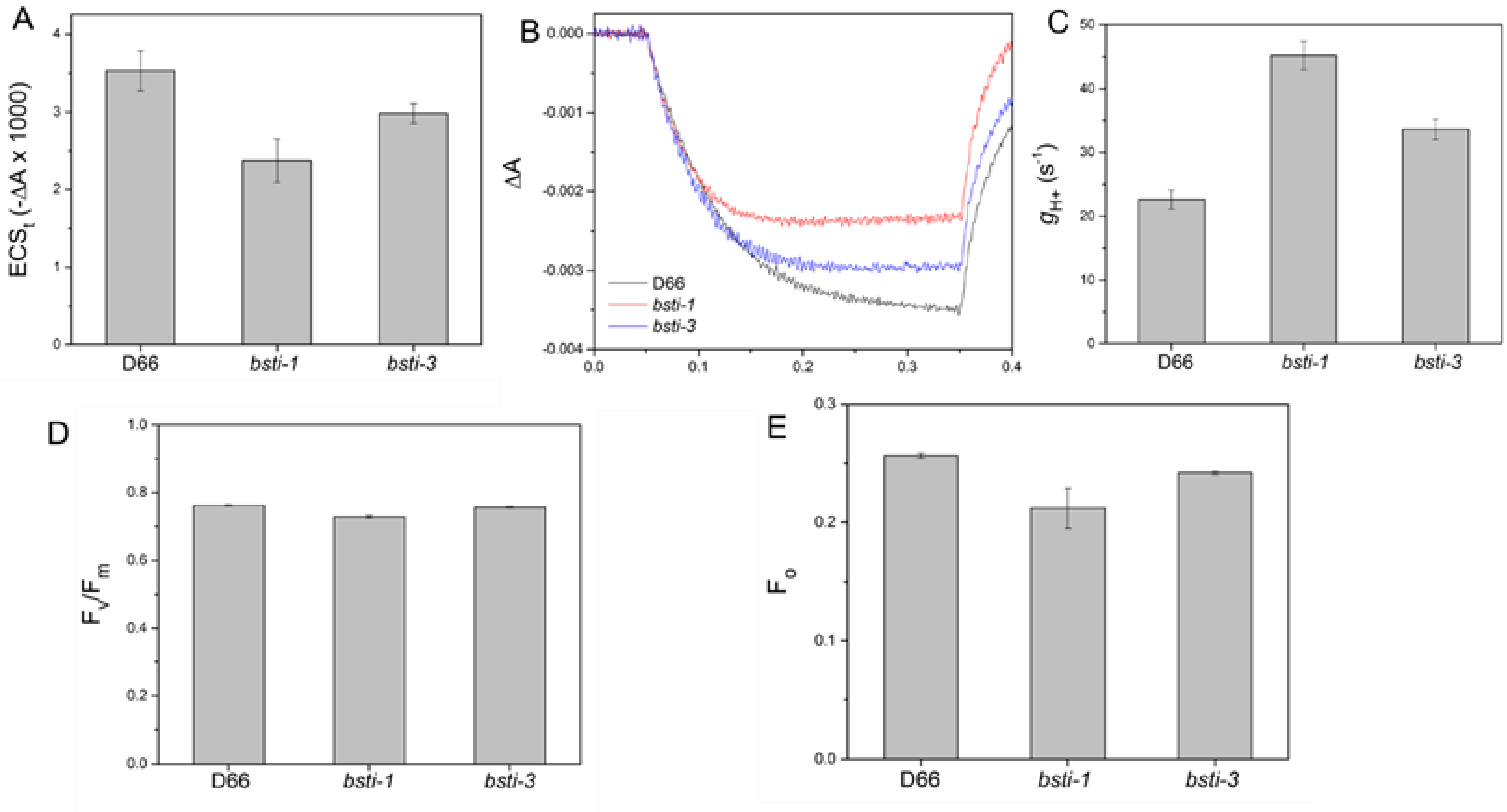


**Fig. S7** Electrochromic shift (ECS) was measured to estimate pmf across the thylakoid membrane (22). (A) pmf was generated by exposing dense cell cultures to actinic light. This light source was switched off from 0.05 to 0.35 sec and carotenoid absorbance was monitored. (B) Total ECS is proportional to the absolute value of maximal ΔA. *bsti* mutants showed less total ECS and therefore have less pmf than D66. (C) The rate at which pmf dissipates is calculated by fitting the ECS curve to a one-component exponential decay function. This time constant is proportional to the proton conductivity of the thylakoid membrane, $g_{H+}$, which is expected to be dominated by ATP synthase activity. *bsti* mutants have higher proton conductivities than D66. (D, E) In a separate experiment using dilute cell cultures, the variable yield of chlorophyll-*a* fluorescence ($F_v/F_m$) was measured to estimate PSII activity. No significant difference in PSII yield (D) or initial fluorescence ($F_o$) (E) was observed.

**Table S1. List of primers used in this study**

| qPCR | Sequence |
|---|---|
| qBST1F | GCTGTGTGGCATTGAGGAGA |
| qBST1R | GGATGAGGCTGATGAGTCCG |
| qBST2F | ACGGTCTACGACTTCCCTCA |
| qBST2R | TTGGATCACGTGGGATTGGG |
| qBST3F | AAGTCAGCAAGGTTCCCTCG |
| qBST3R | TGAATGAGCCTAGCGGGTTG |
| CBLP F | ATGTGCTGTCCGTGGCTTTC |
| CBLP R | CAGACCTTGACCATCTTGTCCC |

| *bst3* Primers | Sequence |
|---|---|
| BST3F | TGCCCCTTCTCAGCACGT |
| BST3R | ACTGCCTCACACTCCCCT |
| CIB1 F | GACGTTACAGCACACCCTTG |
| CIB1 R | GCACCAATCATGTCAAGCCT |
| BST1 RT-F | GACACCAAGACCATCCTGGC |
| BST1 RT-R | AACAGAACTGCAGAGGTCCCG |
| BST2 RT-F | CGGTGCCCATGAGCTCC |
| BST2 RT-R | GCCACTAACCGGCCCAA |
| BST3 RT-F | AATCCCGTCCATGTCGCT |
| BST3 RT-R | CGGCTTGTGAGGACCTCG |
| Actin F | GCCAGAAGGACTCGTACGTT |
| Actin R | CGCCAGAGTCCAGCACGATA |

| Venus Primers | Sequence |
| --- | --- |
| BST1F | GCTACTCACAACAAGCCCAGTTATGCAGATGCAAGCAAACCGTTCGTC |
| BST1R | GAGCCACCCAGATCTCCGTTCTTGCGCTCCCCACCCATGG |
| BST2F | GCTACTCACAACAAGCCCAGTTATGGCCACTGGTCAGACC |
| BST2R | GAGCCACCCAGATCTCCGTTTCTCCTTGTCTCCGCAC |
| BST3F | GCTACTCACAACAAGCCCAGTTATGCAAGTCAGCAAGGTTCCCTCG |
| BST3R | GAGCCACCCAGATCTCCGTTCCGGGGCGAGATGCGCAC |

| miRNA | Sequence |
| --- | --- |
| B2F | CTAGTGAGAGCGTGTTGCAAGGCATATCTCGCTGATCGGCAC<br>CATGGGGGTGGTGGTGATCAGCGCTATATGTTTTGCAACACG<br>CTCTCG |
| B2R | CTAGCGAGAGCGTGTTGCAAAACATATAGCGCTGATCACCAC<br>CACCCCCATGGTGCCGATCAGCGAGATATGCCTTGCAACACG<br>CTCTCA |
| B1F | CTAGTGGGAGCGAGTTGCAAGGCATATCTCGCTGATCGGCAC<br>CATGGGGGTGGTGGTGATCAGCGCTATATGTTTTGCAACTCG<br>CTCCG |
| B1R | CTAGCGGGAGCGAGTTGCAAAACATATAGCGCTGATCACCAC<br>CACCCCCATGGTGCCGATCAGCGAGATATGCCTTGCAACTCG<br>CTCCCA |

**Supplemental References**


1. Ma Y, Pollock SV, Xiao Y, Cunnusamy K, & Moroney JV (2011) Identification of a novel gene, CIA6, required for normal pyrenoid formation in *Chlamydomonas reinhardtii*. *Plant Physiol* 156:884-896.
2. Zhang R, *et al.* (2014) High-throughput genotyping of green algal mutants reveals random distribution of mutagenic insertion sites and endonucleolytic cleavage of transforming DNA. *Plant Cell* 26:1398-1409.
3. Li X, *et al.* (2016) An Indexed, Mapped Mutant Library Enables Reverse Genetics Studies of Biological Processes in *Chlamydomonas reinhardtii*. *Plant Cell* 28:367-387.
4. Sueoka N (1960) Mitotic replication of deoxyribonucleic acid in *Chlamydomonas Reinhardi*. *Proc Natl Acad Sci U S A* 46:83-91.
5. Moroney JV, Husic HD, & Tolbert NE (1985) Effect of carbonic anhydrase inhibitors on inorganic carbon accumulation by *Chlamydomonas reinhardtii*. *Plant Physiol* 79:177-183.
6. Machingura MC, *et al.* (2017) Identification and characterization of a solute carrier, CIA8, involved in inorganic carbon acclimation in *Chlamydomonas reinhardtii*. *J Exp Bot* 68:3879-3890.
7. Molnar A, *et al.* (2009) Highly specific gene silencing by artificial microRNAs in the unicellular alga *Chlamydomonas reinhardtii*. *Plant J* 58:165-174.
8. Shimogawara K, Fujiwara S, Grossman A, & Usuda H (1998) High-efficiency transformation of *Chlamydomonas reinhardtii* by electroporation. *Genetics* 148:1821-1828.
9. Wittkopp TM, *et al.* (2018) GreenCut protein CPLD 49 of *Chlamydomonas reinhardtii* associates with thylakoid membranes and is required for cytochrome b6f complex accumulation. *Plant J* 94:1023-1037.
10. Schindelin J, *et al.* (2012) Fiji: an open-source platform for biological-image analysis. *Nature methods* 9:676.
11. Pettersen EF, *et al.* (2004) UCSF Chimera—a visualization system for exploratory research and analysis. *J Comput Chem.* 25:1605-1612.
12. Benkert P, Tosatto SC, & Schomburg D (2008) QMEAN: A comprehensive scoring function for model quality assessment. *Proteins* 71:261-277.
13. Waterhouse A, *et al.* (2018) SWISS-MODEL: homology modelling of protein structures and complexes. *Nucleic Acids Research* 46:W296-W303.
14. Benson D, Lipman DJ, & Ostell J (1993) GenBank. *Nucleic Acids Res* 21:2963-2965.
15. Goodstein DM, *et al.* (2012) Phytozome: a comparative platform for green plant genomics. *Nucleic Acids Res* 40:D1178-1186.
16. Kearse M, *et al.* (2012) Geneious Basic: an integrated and extendable desktop software platform for the organization and analysis of sequence data. *Bioinformatics* 28:1647-1649.
17. Thompson JD, Higgins DG, & Gibson TJ (1994) CLUSTAL W: improving the sensitivity of progressive multiple sequence alignment through sequence weighting, position-specific gap penalties and weight matrix choice. *Nucleic Acids Res* 22:4673-4680.
18. Henikoff S & Henikoff JG (1992) Amino acid substitution matrices from protein blocks. *Proc Natl Acad Sci U S A* 89:10915-10919.

19. Kumar S, Tamura K, & Nei M (1994) MEGA: Molecular Evolutionary Genetics Analysis software for microcomputers. *Comput Appl Biosci* 10:189-191.
20. Le SQ & Gascuel O (2008) An improved general amino acid replacement matrix. *Mol Biol Evol* 25:1307-1320.
21. Kuhlgert S, *et al.* (2016) MultispeQ Beta: a tool for large-scale plant phenotyping connected to the open PhotosynQ network. *R Soc Open Sci* 3:160592.
22. Cruz JA, *et al.* (2004) Plasticity in light reactions of photosynthesis for energy production and photoprotection. *J Exp Bot* 56:395-406.
